# Region-specific immune regulation determines differential response to JAK1 inhibition in Crohn’s disease *ex vivo*

**DOI:** 10.1093/ecco-jcc/jjag101

**Published:** 2026-07-30

**Authors:** Klaudia Maria Grieger, Susann Dehmel, Valerie Schröder, Kevin Schmidt, Victoria Neufeldt, Laura Bock, Dirk Schaudien, Ulf Kulik, Alexander Wagner, Benjamin Gundert, Heiko Aselmann, Vanessa Neuhaus, Armin Braun, Katherina Sewald

**Affiliations:** Department for Preclinical Pharmacology, Fraunhofer Institute for Toxicology and Experimental Medicine, Hannover, 30625, Germany; Member of the German Center for Lung Research (DZL), Biomedical Research in Endstage and Obstructive Lung Disease Hannover (BREATH) Research Network, Hannover, 30625, Germany; Member of the Fraunhofer Excellence Cluster of Immune Mediated Diseases (CIMD), Germany; Department for Preclinical Pharmacology, Fraunhofer Institute for Toxicology and Experimental Medicine, Hannover, 30625, Germany; Member of the German Center for Lung Research (DZL), Biomedical Research in Endstage and Obstructive Lung Disease Hannover (BREATH) Research Network, Hannover, 30625, Germany; Member of the Fraunhofer Excellence Cluster of Immune Mediated Diseases (CIMD), Germany; Department for Preclinical Pharmacology, Fraunhofer Institute for Toxicology and Experimental Medicine, Hannover, 30625, Germany; Member of the German Center for Lung Research (DZL), Biomedical Research in Endstage and Obstructive Lung Disease Hannover (BREATH) Research Network, Hannover, 30625, Germany; Member of the Fraunhofer Excellence Cluster of Immune Mediated Diseases (CIMD), Germany; Department for Preclinical Pharmacology, Fraunhofer Institute for Toxicology and Experimental Medicine, Hannover, 30625, Germany; Member of the German Center for Lung Research (DZL), Biomedical Research in Endstage and Obstructive Lung Disease Hannover (BREATH) Research Network, Hannover, 30625, Germany; Member of the Fraunhofer Excellence Cluster of Immune Mediated Diseases (CIMD), Germany; Department for Preclinical Pharmacology, Fraunhofer Institute for Toxicology and Experimental Medicine, Hannover, 30625, Germany; Department for Preclinical Pharmacology, Fraunhofer Institute for Toxicology and Experimental Medicine, Hannover, 30625, Germany; Department for Preclinical Pharmacology, Fraunhofer Institute for Toxicology and Experimental Medicine, Hannover, 30625, Germany; Department for General, Visceral and Transplantation Surgery, Hannover Medical School (MHH), Hannover, 30625, Germany; Department for General, Visceral and Transplantation Surgery, Hannover Medical School (MHH), Hannover, 30625, Germany; Clinic for General, Visceral and Minimally Invasive Surgery, KRH Klinikum Region Hannover, Hannover, 30459, Germany; Clinic for General, Visceral and Minimally Invasive Surgery, KRH Klinikum Region Hannover, Hannover, 30459, Germany; Department for Preclinical Pharmacology, Fraunhofer Institute for Toxicology and Experimental Medicine, Hannover, 30625, Germany; Member of the German Center for Lung Research (DZL), Biomedical Research in Endstage and Obstructive Lung Disease Hannover (BREATH) Research Network, Hannover, 30625, Germany; Member of the Fraunhofer Excellence Cluster of Immune Mediated Diseases (CIMD), Germany; Department for Preclinical Pharmacology, Fraunhofer Institute for Toxicology and Experimental Medicine, Hannover, 30625, Germany; Member of the German Center for Lung Research (DZL), Biomedical Research in Endstage and Obstructive Lung Disease Hannover (BREATH) Research Network, Hannover, 30625, Germany; Member of the Fraunhofer Excellence Cluster of Immune Mediated Diseases (CIMD), Germany; Department for Preclinical Pharmacology, Fraunhofer Institute for Toxicology and Experimental Medicine, Hannover, 30625, Germany; Member of the German Center for Lung Research (DZL), Biomedical Research in Endstage and Obstructive Lung Disease Hannover (BREATH) Research Network, Hannover, 30625, Germany; Member of the Fraunhofer Excellence Cluster of Immune Mediated Diseases (CIMD), Germany

**Keywords:** precision-cut intestinal slices, Crohn’s disease, filgotinib, T cell, *ex vivo*, immunity

## Abstract

Crohn’s disease (CD) remains a significant therapeutic challenge, with limited treatment options and heterogeneous clinical responses. The selective JAK1 inhibitor filgotinib has shown great potential in treating ulcerative colitis but failed to demonstrate consistent efficacy in CD, for reasons that remain unclear. Here, we address this gap using an immunocompetent *ex vivo* model of precision-cut intestinal slices (PCIS) from CD patients, allowing region-specific analysis of ileal and colonic responses. We reveal that colonic PCIS display strong T cell activation and are highly sensitive to filgotinib, with suppression of Th1/Th17 cytokines, oxidative stress, and STAT signaling *ex vivo*. In contrast, ileal PCIS exhibited predominantly innate and Th17-driven inflammation, and were less sensitive to JAK1 inhibition. These data provide a potential mechanistic explanation for the limited efficacy of filgotinib in CD clinical trials and established PCIS as a human-relevant *ex vivo* model for region-specific drug responses, underscoring the importance of developing therapies tailored to different segments of the gut in inflammatory bowel disease.

## 1. Introduction

Inflammatory bowel diseases (IBD), including Crohn’s disease (CD) and ulcerative colitis (UC), are chronic, relapsing–remitting disorders of the gastrointestinal tract that require lifelong management and are associated with substantial morbidity.^1^^,^^2^ CD is clinically and immunologically heterogeneous and can affect any part of the gastrointestinal tract and differs fundamentally from UC in its transmural, segmental inflammation, often affecting the terminal ileum and proximal colon.^3–5^ Roughly one-third of patients present with isolated ileitis, while another third show ileocolonic involvement, and a smaller subset exhibit isolated colonic disease.^6^^,^^7^ This anatomical variation is increasingly recognized as a determinant of therapeutic efficacy, yet the mechanistic basis for region-dependent drug responses remains unresolved.^8^^,^^9^ Distinct immune and pathophysiological features of ileal versus colonic CD have been demonstrated at proteomic, transcriptomic, and single-cell levels.^10–15^ Importantly, region-dependent efficacy has been suggested for several therapies, but most evidence derives from post-hoc analysis rather than dedicated mechanistic studies.^16–19^ However, whether these baseline regional differences translate into differential responsiveness to targeted therapies has not been addressed in a dedicated human tissue setting.

The standard care for CD includes corticosteroids (e.g., prednisolone),^20^^,^^21^ immunosuppressants (e.g., azathioprine and methotrexate),^22^ and biologics targeting tumor necrosis factor (TNF), integrins, or IL-23.^23–26^ Despite these established treatments, up to 60% of patients fail to achieve or sustain remission.^27–29^ As a newer class of orally available small molecules, Janus kinase (JAK) inhibitors have emerged as promising therapeutic agents targeting central pro-inflammatory pathways.^30^^,^^31^ The JAK family involves JAK1, JAK2, JAK3, and TYK2, which interact with specific cytokine receptors to initiate signal transducer and activator of transcription (STAT) phosphorylation.^32^ This ultimately drives the transcriptional activation of immune effector genes, such as *IFNG* or *TNF.*^32^ Filgotinib, a selective JAK1 inhibitor, is approved for UC but failed to show efficacy in CD in the DIVERSITY phase III trial.^33^^,^^34^ This discrepancy raises a key mechanistic question as to whether inflammatory programs in ileal and colonic CD are equally dependent on JAK1 signaling. Given the known regional heterogeneity of CD, differential segment-specific sensitivity to JAK1 inhibition represents a plausible explanation for the limited efficacy of filgotinib in CD.

Here, we directly tested whether ileal and colonic CD differ in their responsiveness to JAK1 inhibition. To achieve this, we used precision-cut intestinal slices (PCIS) from patient-derived human tissue, which preserves native tissue architecture and multicellular immune complexity *ex vivo*. We hypothesized that regional immune programs in terminal ileum and colon differ in their dependence on JAK1 signaling and therefore respond differently to filgotinib. By systematically comparing immune activation and drug effects in terminal ileum and ascending colon, we aimed to define a mechanistic basis for region-specific treatment response in CD. In addition, this approach allowed us to assess the value of PCIS as a human translational model for segment-specific drug profiling in IBD *ex vivo*.

## 2. Methods

### 2.1. Ethical statement

The utilization of human intestinal tissue in experiments was approved by the Ethics Committee of Hannover Medical School, Lower Saxony Medical Association for Siloah hospital Hannover and complies with the World Medical Association’s Code of Ethics (updated November 1, 2024, reference number 3082-2016). All patients or their relatives, caregivers, or guardians provided written informed consent for the use of intestinal tissue for research.

### 2.2. Preparation and cultivation of PCIS

PCIS were generated from intestinal tissue of female and male patients undergoing surgical resection for CD (mean age 46 ± 13 years) or cancer resection (non-CD; mean age 70 ± 7.5 years; Table 1; Table S1). In CD patients, resection was performed in cases of severe disease, often accompanied by complications such as strictures, fistulas, or an insufficient response to medical treatment. For controls without CD (non-CD), only healthy parts of the tissue from resection margins were used. PCIS from CD patients were generated exclusively from visibly inflamed areas with confirmed Crohn’s diagnosis. Samples were included only if they showed high IL-8 expression, indicating active inflammation; this criterion applied to all CD cases, regardless of ileal or colonic resection type. The preparation and cultivation of PCIS were performed according to an adapted protocol by de Graaf et al. 2010,^35^ as described also in a previous publication.^36^ Colon and ileum tissues were processed and cultured using the same protocol. After removing the muscle layers and residual fat, mucosal sheets were embedded in 3% low-melting agarose (Fisher, 10583355). PCIS were prepared using a microtome (Krumdieck Tissue Slicer) to generate tissue slices of approximately 300 µm thickness. PCIS were cultured in 12-well plates in WME medium (Gibco, 32551020) with 14 mM d-glucose (Sigma, C8270) and 1× Antibiotic-Antimycotic (Gibco, 15240062) at 37 °C, 95% O_2_/5% CO_2_, with shaking at 90 rpm. PCIS were cultured for 24 h with or without treatment. Immune cell stimulation was performed using either 10 µg/mL Concanavalin A from *Canavalia ensiformis* (ConA, Sigma, C5275) or 10 µL/mL ImmunoCult^TM^ Human CD3/CD28 T cell activator (anti-CD3/CD28, STEMCELL Technologies, 10971). To inhibit JAK1 signaling, filgotinib (Filg, MCE, HY-18300) was applied at concentrations of 1 - 100 nM, and DMSO (0.05%) was included as a corresponding vehicle (Veh) control. Each experiment was set up in technical duplicates per donor (two wells with two PCIS per well).

**Table 1.** Patients’ demographic and diagnosis details.

Gender	Age (years)	Diagnosis	Removed segment	Classification
**Male**	78	Cecum carcinoma	Terminal ileum	non-CD
**Male**	76	Colon carcinoma	Terminal ileum	non-CD
**Female**	75	Colon carcinoma	Terminal ileum	non-CD
**Female**	64	Colon carcinoma	Transverse colon	non-CD
**Female**	76	Liver cirrhosis, colon carcinoma	Terminal ileum & ascending colon	non-CD
**Female**	58	Ascending colon carcinoma	Terminal ileum & ascending colon	non-CD
**Male**	69	Adenocarcinoma	Ascending colon	non-CD
**Female**	41	Crohn’s disease	Terminal ileum & ascending colon	CD
**Male**	62	Crohn’s disease	Terminal ileum & ascending colon	CD
**Female**	62	Crohn’s disease	Neoterminal ileum & ascending colon	CD
**Female**	40	Crohn’s disease	Terminal ileum	CD
**Male**	46	Crohn’s disease	Terminal ileum & ascending colon	CD
**Female**	29	Crohn’s disease	Terminal ileum	CD

### 2.3. Viability assay

The viability of PCIS was determined by lactate dehydrogenase (LDH) release assays from supernatants (LDH Cytotoxicity Assay Kit, Roche, 11644793001) according to the manufacturer’s instructions. Triton X-100 (1% in phosphate-buffered saline [Sigma])-treated PCIS were investigated as a reference (dead) control.

### 2.4. Histopathology

Cultured PCIS were fixed in 10% neutral buffered formalin for 24 h, embedded in paraffin and sliced into 4-µm sections. The paraffin sections were stained with hematoxylin and eosin (H&E) using standard histological procedures, then mounted for light microscopy. Magnification and scale bars are indicated in the figures. Samples from *N* = 4 donors each for ileal and colonic CD-derived PCIS, with two slices per donor, were stained.

### 2.5. Whole-mount immunofluorescence

CD- and non-CD-derived PCIS from human colon and ileum samples were fixed overnight in 2% PFA and subsequently washed with PBS. Samples from *N* = 3 donors each for ileal and colonic PCIS, with two slices per donor, were stained. Primary antibodies included anti-occludin mouse monoclonal antibody (6.6 µg/mL, OC-3F10 clone, Invitrogen, 33-1500 mouse IgG1 κ isotype) and anti-ZO-1 rabbit polyclonal antibody (3.3 µg/mL, Invitrogen, 61-7300, rabbit IgG isotype). Secondary antibodies were applied at a dilution of 1:200: AlexaFluor^®^ 568-conjugated goat anti-rabbit IgG H&L (Abcam, ab175471) and AlexaFluor^®^ 647-conjugated goat anti-mouse IgG H+L (Jackson ImmunoResearch, 115-605-003). Nuclear staining was performed with 4′,6-diamidino-2-phenylindole (DAPI, Sigma, D9542) for 30 min. Stained PCIS were embedded in ibidi mounting medium (ibidi, 50001) for subsequent confocal microscopy. Z-stacks of 25 µm were imaged every 0.45 µm with a Zeiss LSM 800 confocal microscope using a 20× water immersion objective. The images were reconstructed in three dimensions using IMARIS^®^ 9.2.1 for the volumetric projection (Bitplane). Quantification was performed using IMARIS software, with analysis parameters optimized independently for ileal and colonic PCIS and applied to all samples. Two regions of interest (ROIs) were selected in the ileum (villous region) and four in the colon (two within crypts and two outside the crypts) for each image. Signal intensity was determined as the total antibody volume and expressed as a sum of volumes (µm^3^).

### 2.6. Analysis of mediator secretion

After 24 h, PCIS culture supernatants were collected, supplemented with a 0.2% protease inhibitor cocktail (Thermo Fisher Scientific, product code 87785), and stored at −80 °C until analysis. A panel of 20 cytokines and chemokines was analyzed in PCIS supernatants with a Mesoscale Discovery customized U-PLEX^®^ assay (Mesoscale Diagnostic) according to the manufacturer’s instructions. The panel included the following analytes (lower limit of quantification as pg/mL): IL-2 (0.2 pg/mL), IL-10 (0.5 pg/mL), TNF-α (0.5 pg/mL), GM-CSF (1.1 pg/mL), IFN-γ (4.1 pg/mL), IL-5 (0.6 pg/mL), IL-17A (2.7 pg/mL), IL-17F (60.1 pg/mL), IL-21 (7.5 pg/mL), IL-22 (0.4 pg/mL), IL-23 (3.8 pg/mL), granzyme A (0.2 pg/mL), granzyme B (0.9 pg/mL), TRAIL (1.1 pg/mL), TSLP (0.9 pg/mL), IL-18 (1.8 pg/mL), IL-33 (1.1 pg/mL), CXCL10 (1.3 pg/mL), MIP-3α (3.1 pg/mL), and IL-12p70 (0.8 pg/mL). Plates were measured with the MESO QuickPlex SQ120 (Mesoscale Diagnostic). Plotted values below the level of quantification were extrapolated using MSD software. Further analytes were measured using DuoSet ELISA kits (R&D, DY206 and DY208) according to the manufacturer’s recommendations (lower limit of detection): human IL-6 (9.4 pg/mL) and IL-8 (31.2 pg/mL).

### 2.7. RNA isolation and mRNA sequencing

For RNA isolation, treated and control PCIS were collected after 24 h of incubation and preserved in RNAlater^TM^ (Invitrogen, AM7021). RNAlater^TM^ was removed following overnight incubation, and the samples were stored at −80 °C. Total RNA was isolated as previously described.^36^^,^^37^ All samples showed high RNA integrity (RIN > 7). mRNA sequencing was performed by Novogene using standard Illumina protocols with poly(A) enrichment. The Illumina (NovaSeq X Plus) platform was used for paired-end 150-bp sequencing (30 million reads per sample).

### 2.8. Analysis of mRNA sequencing

Fastp was utilized for quality control of raw sequencing reads prior to alignment to the *Homo sapiens* reference genome (GRCh38/hg38) with STAR.^38^^,^^39^ Reads per gene were counted with the “GeneCounts” quantification mode implemented in STAR based on ENSEMBL human transcript reference assembly v.113. All genes with a sum of counts across all samples less than the number of samples were excluded from further analyses. Subsequently, the DESeq2 package^40^ (v.1.46.0) for R (v.4.4.1) was used to normalize raw counts and perform differential gene expression calculations with default parameters except “local” fit type was used for dispersion estimation. Genes with an adjusted *P-*value of <.05 and an absolute log_2_ fold change (log_2_FC) ≥ 1 were considered significantly differentially expressed. Over-representation analyses (ORA) in significant differentially expressed genes (DEGs) were performed using the gprofiler2 (v.0.2.3) package for R to identify significantly enriched biological pathways and processes from the Gene Ontology (GO), KEGG, WikiPathways, and Reactome databases. Venn diagrams were created using the results of the DEGs and ORA.^41^

### 2.9. Analysis of reactive oxygen/nitrogen species

A DCF reactive oxygen/nitrogen species (ROS/RNS) assay (Abcam, ab238535) was used to quantify the release of ROS/RNS in the supernatant of CD-derived PCIS from ileum and colon after 24 h of cultivation with or without treatments. The assay was performed according to the manufacturer’s protocol. Undiluted cell-free supernatant was incubated with catalyst for 5 min at room temperature, followed by incubation with 2′,7′-dichlorodihydrofluorescein (DCFH) solution in the dark for 15 min. Fluorescence intensity at 535 nm (λ_Excitation_ = 485 nm) was measured using a microplate reader (Microplate Reader Infinite^®^ 200 Pro Tecan Group, Männedorf, Switzerland). The assay was considered valid if a positive signal for 20 µM H_2_O_2_ was detected. Mean fluorescence intensity (MFI) values were normalized to the total protein content of the analyzed samples, measured using a Pierce^TM^ BCA protein assay kit (Thermo Fisher, 23225) according to the manufacturer’s instructions. Protein content was determined from the same PCIS samples after completion of the assay by lysing the corresponding tissue slices and measuring total protein using the BCA assay kit. Although PCIS were prepared at standardized thickness (∼300 µm), minor variation in slice size and cellular composition occurs. Therefore, normalization to total protein was applied to reduce technical variability between slices. This normalization follows the assay recommendations and was applied to ROS/RNS and nitrogen oxides (NOx) measurements.

### 2.10. Analysis of dissolved nitrogen oxides

For the determination of nitrate (NO_3_^−^) and nitrite (NO_2_^−^) levels in supernatants of CD-derived PCIS from human ileum and colon tissue, the nitric oxide assay kit (Invitrogen, EMSNO) was utilized according to the manufacturer’s instructions. In total, 250 µL (diluted 1:2 with reagent diluent) of cell-free supernatants were centrifuged through an ultracentrifugation filter (pierce concentrators) at 15 000 *g* for 30 min at room temperature and 50 µL in duplicates were used immediately for each assay. Separate plates for the measurement of nitrite and nitrate were prepared and followed the instructions. The optical density was measured at 540 nm using a microplate reader (Microplate Reader Infinite^®^ 200 Pro Tecan Group). Individual nitrate und nitrite values were added to yield total NOx in ng/mL and normalized to the total protein content of the analyzed samples, measured using a Pierce^TM^ BCA protein assay kit (Thermo Fisher, 23225) according to the manufacturer’s instructions.

### 2.11. Statistical analysis

Statistical analyses were performed with GraphPad Prism (v.10.1.24). Unpaired two-tailed *t*-tests were performed to compare two groups. Two-way ANOVA was performed to evaluate the effects of two factors (segment and treatment). Sidak’s multiple comparison test was applied in all ANOVA tests. Differences were considered significant at *P* < .05 (*), 0.01 (**), 0.001 (***), or 0.0001 (****). All data were from a minimum of three independent experiments.

## 3. Results

### 3.1. Distinct baseline immune programs in ileal and colonic CD-derived PCIS provide a framework for region-specific response to JAK1 inhibition

Based on our previous work showing that PCIS preserve disease-related immune features *ex vivo*,^36^ we next asked whether ileal and colonic CD-derived PCIS differ in baseline inflammatory programs in a manner that may predict differential responsiveness to JAK1 inhibition.

CD-derived PCIS retained key disease-associated characteristics *ex vivo*. Basal release of the proinflammatory mediators IL-8 and IL-6 was increased in CD-derived PCIS compared with region-matched non-CD controls, whereas LDH release remained comparable, indicating preserved tissue viability under basal culture conditions (Figure S1). To further characterize epithelial integrity, whole-mount immunofluorescence staining for the tight junction proteins ZO-1 and occludin was performed in ileal and colonic PCIS from CD and non-CD patients. In ileal PCIS, CD-derived tissue showed reduced ZO-1 and occludin staining compared with non-CD controls, consistent with impaired epithelial barrier architecture, whereas no significant differences were detected in colonic PCIS under these experimental conditions (Figures S2 and S3). Together, these data demonstrate that PCIS preserve inflammatory and epithelial disease-associated features *ex vivo* and provide the pathological context for the subsequent analysis of region-specific immune responses and filgotinib effects.

**Figure 1.**
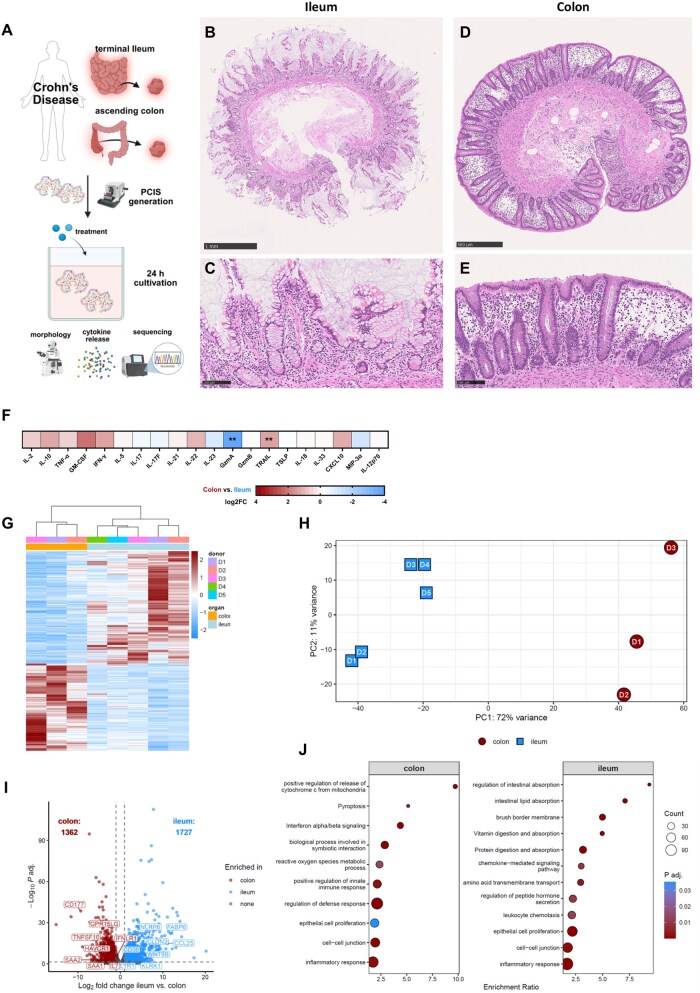
Region-specific functional and transcriptional profiles are maintained *ex vivo* in terminal ileum and ascending colon precision-cut intestinal slices (PCIS) from Crohn’s disease (CD) patients. (A) Schematic representation of the experimental workflow of PCIS generation and analysis (created with BioRender.com). Hematoxylin & eosin staining of formalin-fixed, paraffin-embedded thin sections (4 μm) of ileum (B, C) and colon (D, E) tissue slices from CD patients after 24 h of cultivation; representative images of *N* = 4 donors. (B, D) Overview images, scale bar=1 mm and (C, E) 20× magnification of selected region, scale bar=100 µm. (F) Unstimulated tissue slices were cultivated in medium for 24 h to determine basal secretion of several mediators in supernatant of colon vs ileum tissue slices from CD patients presented as Log_2_ normalized fold changes (log_2_FC > 0 values [red]: higher in colon, < 0 values [blue]: higher in ileum). *N* = 4 colon and *N* = 6 ileum donors (two technical replicates per donor) were analyzed. *P < .05, **P < .01 by unpaired two tailed *t*-test between colon and ileum. (G–J) Region-specific transcriptional differences between PCIS derived from colon (*N* = 3) and ileum (*N* = 5) of CD patients were analyzed by mRNA sequencing. (G) Heatmap displaying expression levels of DEGs (adj. *P* < .05, |log2[FC] | ≥ 1) among investigated groups. Hierarchical clustering based on Euclidean distance. (H) Principial component analysis (PCA) based on the complete transcriptome-wide dataset showing separation of ileal and colonic CD-derived PCIS samples. Statistical significance of group separation was assessed by PERMANOVA (adonis2) on variance-stabilized count data using Euclidean distances. (I) Volcano plot highlighting differential expression of genes in CD-derived PCIS comparing colon and ileum. Red: genes enriched in colon, blue: genes enriched in ileum, grey non significant genes (differentially expressed genes [DEGs]; adj. *P* < .05, |log2[FC] | ≤ −1 for colon or ≥ 1 for ileum). (J) Over-representation analysis (ORA) of significantly enriched biological pathways based on DEGs identified in colon and ileum PCIS from CD patients.

PCIS prepared from ileum and colon resections of CD patients preserved expected region-specific architecture *ex vivo*, showing prominent finger-like villi in the ileum and larger crypts structures in the colon. Key mucosal components were preserved in both regions, including epithelial cells, immune cells in the lamina propria, and the underlying muscularis mucosae (Figure 1A–E).

At baseline, CD-derived PCIS already exhibited marked region-specific immune profiles. Ileal PCIS showed increased abundance of secreted mediators in the supernatant, including MIP-3α and GzmA, indicative of innate and epithelial-associated inflammation. In contrast, colonic PCIS displayed higher levels of secreted mediators, including GM-CSF, IFN-γ, and TRAIL, consistent with a Th1/cytotoxic immune profile (Figure 1F).

Moreover, we performed mRNA sequencing in CD-derived PCIS samples to obtain a comprehensive overview of transcriptional differences between ileal and colonic regions. As expected for two anatomically distinct intestinal segments, principal component analysis **(**PCA) of global gene expression profiles showed clear separation of ileal and colonic samples across all donors, indicating preservation of region-specific transcriptional identity *ex vivo* (Figure 1G–H). This separation was statistically supported by PERMANOVA performed on variance-stabilized count data using Euclidean distances (*P* = .013). Direct comparison between the two anatomical regions identified 1727 protein-coding genes enriched in the ileum and 1362 enriched in the colon. In line with the cytokine profiles, ileal PCIS were enriched for genes linked to innate and barrier-associated inflammatory pathways, including *CCL25*, *IL1R1*, *NLRP6*, and *KLRK1*. In contrast, colonic PCIS showed enrichment of genes associated with Th1/cytotoxic and T cell-related responses, including *TNFSF10* (gene coding for TRAIL protein), *IFNLR1*, *GPR15LG*, and *IL-7* (Figure 1I).

To obtain an overview of biological processes and pathways associated with each intestinal region, genes identified as differentially abundant in the direct comparison between ileal and colonic CD-derived PCIS were subjected to over-representation analysis using the GO, KEGG, WikiPathways, and Reactome databases (Table S2). Enrichment analysis was performed separately for ileum-enriched and colon-enriched gene sets, thereby capturing region-specific functional characteristics (Figure 1J). Ileal CD-derived PCIS showed enrichment in pathways related to absorption and nutrient transport, while colonic CD-derived PCIS showed enrichment in symbiotic interactions, reactive oxygen species (ROS) metabolism, epithelial proliferation, and cell junctions. Both regions showed upregulation of proinflammatory pathways, including pyroptosis and interferon signaling in the colon, and leukocyte chemotaxis in the ileum (Figure 1J). Together, these data indicated that ileal and colonic CD-derived PCIS differ in their baseline inflammatory architecture and suggested that their response to JAK1 inhibition may also differ.

### 3.2. Colonic CD-derived PCIS display stronger inducible T cell programs, whereas ileal PCIS show broader innate-inflammatory reactivity

**Figure 2.**
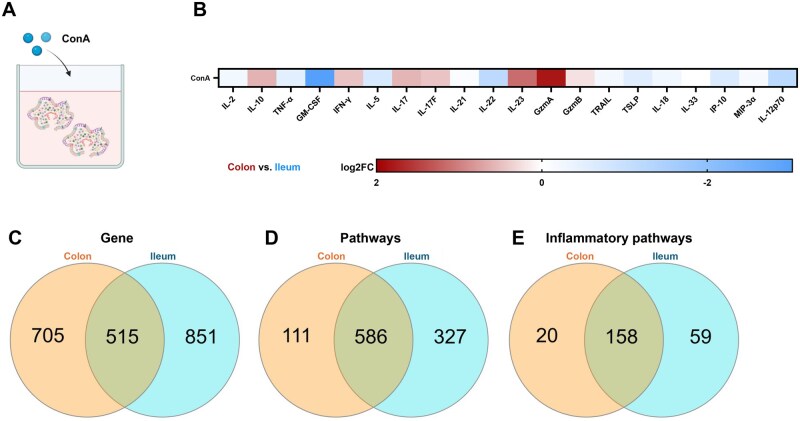
Differential activation of inflammatory and T cell signaling in terminal ileum versus ascending colon of Crohn’s disease (CD) patients *ex vivo*. Colon or ileum tissue slices from CD patients were cultivated *ex vivo* either unstimulated (Med) or stimulated with 10 μg/mL Concanavalin A (ConA). Mediator secretion and gene expression were analyzed. Fold changes represent stimulation-induced responses and therefore reflect inducible immune capacity rather than baseline regional abundance. (A) Schematic representation (created with BioRender.com). (B) Released mediator levels as Log_2_ fold changes (colon vs ileum) of stimulation-induced fold changes (medium [Med] vs ConA stimulation) in precision-cut intestinal slices (PCIS) from CD patients. *N* = 4 colon and *N* = 6 ileum donors (log_2_FC, > 0 values [red]: higher in colon, < 0 values [blue]: higher in ileum). A minimum of two technical replicates (two wells with two tissue slices each) were analyzed per donor. (C–E) Gene expression analysis after ConA stimulation, comparing colon and ileum PCIS from CD patients. *N* = 3 colon and *N* = 5 ileum donors. Venn-diagrams highlighting the overlap of all significantly differentially regulated genes (C, adj. *P* < .05), all pathways (D), and inflammatory pathways (E) by comparing colon and ileum CD-derived PCIS after ConA stimulation. Complete lists of differentially expressed genes and pathway enrichment results, including statistical parameters and contributing genes for each enriched term, are provided in Supplementary Tables S3–S5. Enriched terms were derived from GO, KEGG, Reactome, and WikiPathways annotations; therefore, individual genes may contribute to multiple enriched terms.

Despite a few studies indicating proteomic and transcriptomic heterogeneity between ileal and colonic CD,^10–13^ region-specific immune responses and functional tissue reactivity have not been systematically investigated. Although CD-derived PCIS already displayed basal inflammatory activity *ex vivo*, these baseline signals primarily reflect the inflammatory state of the tissue at the time of resection and may be influenced by donor-specific disease activity, tissue composition, and sampling variability. To assess the inducible inflammatory capacity and functional response of each intestinal region under standardized conditions, we therefore applied two complementary immune challenges. ConA was used as a broad polyclonal stimulus to activate resident immune cells within the preserved tissue context, whereas anti-CD3/CD28 stimulation served as a more T cell-directed stimulatory condition. These stimulation conditions were not intended to mimic the basal physiological state of CD tissue, but to reveal region-specific inducible immune programs and to establish a dynamic range for subsequent pharmacodynamic testing of JAK1 inhibition. To determine whether these baseline differences translate into distinct functional immune responsiveness, ileal and colonic CD-derived PCIS were stimulated *ex vivo* with ConA or anti-CD3/CD28 and analyzed for cytokine release and transcriptional responses after 24 h. Neither stimulus affected tissue viability (Figure S4A). Following ConA or anti-CD3/CD28 stimulation, distinct region-specific cytokine responses were observed (Figure 2B; Figures S5 and S6B). Ileal CD-derived PCIS showed increased release of proinflammatory cytokines, including TNF-α (log_2_FC: −0.52 | −1.85; ConA | aCD3/CD28) and GM-CSF (log_2_FC: −3.03 | −4.2), compared with colonic CD-derived PCIS, in response to both stimuli. In contrast, colonic CD-derived PCIS revealed increased inducibility of T cell-associated mediators including IFN-γ (log_2_FC: 0.47 | 1.20), IL-17F (log_2_FC: 0.46 | 0.51), IL-23 (log2FC: 1.14 | 1.41), and GzmA (log_2_FC: 1.82 | 1.41), compared wtih ileal CD-derived PCIS under both stimulations. These stimulation-induced patterns should be interpreted separately from the baseline comparison shown in Figure 1F. Figure 2B illustrates stimulation-induced responses and should be interpreted as a functional immune challenge rather than as a representation of the basal inflammatory state shown in Figure 1. Accordingly, mediators may differ between ileum and colon at baseline but show a different or even opposite regional pattern after stimulation. For example, GM-CSF showed higher relative abundance in colonic PCIS at baseline, but stronger ConA-induced responsiveness in ileal PCIS, indicating that basal inflammatory state and inducible immune capacity represent distinct layers of region-specific immune regulation.

Transcriptomic analysis following ConA stimulation revealed 2071 significantly regulated genes, with 515 shared, 851 ileum-specific, and 705 colon-specific genes in CD-derived PCIS (Figure 2C; Table S3). Euclidean distance-based clustering provided an overview of ConA-induced transcriptional changes and showed that baseline differences between ileal and colonic CD-derived PCIS remained apparent after stimulation (Figure S4B). DEGs in ileal and colonic samples were subsequently used for separate over-representation analyses (Table S4). This analysis identified a substantial number of enriched functional terms shared between ileal and colonic PCIS, indicating that both tissue regions activate common inflammatory programs upon ConA stimulation. In addition, region-enriched functional terms were detected, with ileal responses showing broader enrichment of innate, mucosal, and pro-inflammatory immune processes, whereas colonic responses included selected T cell-associated and canonical NF-κB-related immune programs (Figure 2D; Table S5). Because individual genes can be annotated to multiple pathways and ontology terms, the number of enriched terms exceeds the number of input genes and should be interpreted as functional annotations.

Notably, the ileum revealed an increased number of proinflammatory pathways than the colon (59 vs 20), reflecting broader activation of both innate and adaptive immunity (Figure 2E; Table S5). Similarly, anti-CD3/CD28 stimulation induced both shared and region-enriched transcriptional programs in ileal and colonic CD-derived PCIS, as reflected by the overlap of differentially regulated genes and pathway enrichment analyses (Figure S4C, Figure S6C-D). Immune pathways related to T cells were predominantly enriched in colonic CD-derived PCIS compared with ileal CD-derived PCIS (37 vs 7), including immature T cell proliferation, Th1 and Th17 differentiation, and memory T cell differentiation. However, ileal CD-derived PCIS showed selective responsiveness marked by Th17-associated gene signatures (Figure S6F).

Thus, *ex vivo* stimulation revealed that colonic CD-derived PCIS are characterized by stronger inducible T cell activation, whereas ileal tissue retains a broader inflammatory program with a substantial innate component. Importantly, however, the strongest regional divergence was observed when ConA-stimulated tissues were treated with filgotinib, indicating that ileal and colonic inflammatory programs differ not only at baseline or upon stimulation, but particularly in their responsiveness to JAK1 inhibition.

### 3.3. Filgotinib suppresses key Th1/Th17 and cytotoxic inflammatory mediators more effectively in colonic than in ileal CD-derived PCIS

Filgotinib, a selective JAK1 inhibitor, has proven clinical efficacy for UC, but lacked sufficient efficacy in clinical trials for CD and was therefore not approved for this indication.^33^^,^^34^ This could be related to underlying immune phenotypes in the colon and ileum.

Given the marked regional differences in baseline and inducible immune programs, we next tested whether responsiveness to JAK1 inhibition also differs between ileal and colonic CD-derived PCIS. Therefore, to improve the interpretability of the regional drug response, we analyzed the effects of filgotinib in a stepwise manner. First, we assessed whether filgotinib suppressed inflammatory mediator release in both intestinal regions. Second, we identified mediators showing preferential or stronger inhibition in colonic compared with ileal PCIS. Finally, we integrated mediator release with transcriptomic and pathway-level analyses to determine whether differential pharmacodynamic responses were reflected by broader region-specific transcriptional programs. JAK/STAT signaling was detectable in both regions, and filgotinib was therefore evaluated under ConA-driven inflammatory conditions *ex vivo*.

**Figure 3.**
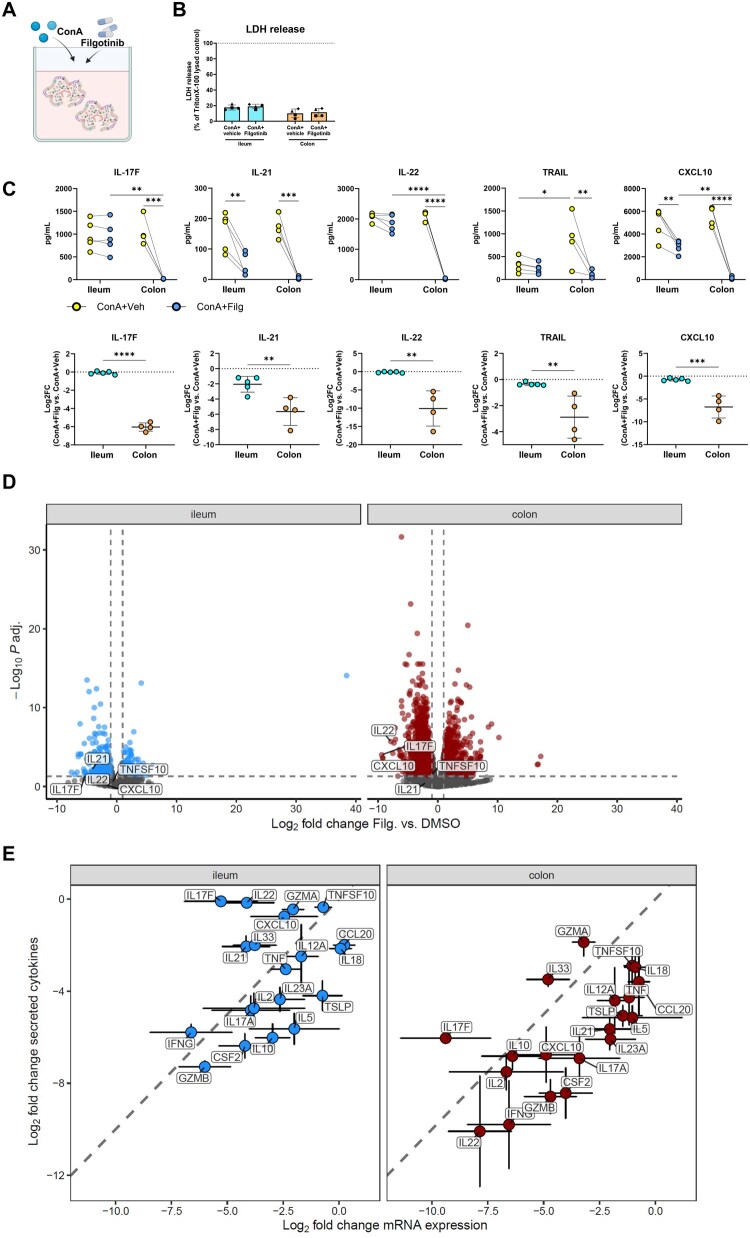
Filgotinib shows better efficacy in colon vs ileum precision-cut intestinal slices (PCIS) from Crohn’s disease (CD) patients in regulating critical type 1 and 17 related responses. Colon or ileum CD-derived PCIS were incubated for 24 h either unstimulated (Med) or stimulated using 10 μg/mL Concanavalin A combined with 100 nM filgotinib (ConA + Filg) or 0.05% DMSO vehicle control (ConA + Veh). Viability, absolute secretion of several mediators in supernatant, and gene expression analyses were performed. (A) Schematic representation (created with BioRender.com). (B) LDH release in supernatant normalized to lactate dehydrogenase (LDH) release of Triton X-100 lysed control tissue slices. (C) Absolute release of IL-17F, IL-21, IL-22, TRAIL, and CXCL10 levels in supernatant. *N* = 4 colon and *N* = 5 ileum donors. *P < .05, **P < .01, ***P < .001, and ****P < .0001 by two-way ANOVA with Sidak’s multiple comparison test comparing ConA + vehicle vs ConA + filgotinib or ileum vs colon. (D) Volcano plot highlighting differential expression of genes in CD-derived PCIS comparing ConA + filgotinib vs ConA + vehicle in colon and ileum with highlighted genes for selected cytokines. It shows selected mediator-associated genes corresponding to the protein mediators highlighted in (C). This targeted analysis was used to assess whether the stronger mediator-level suppression observed in colonic PCIS was reflected at the mRNA level. The complete list of differentially expressed genes (DEGs) is provided in Table S6. Blue: enriched genes in ileum, red: enriched genes in colon, grey: no significant genes (DEGs; adj. *P* < .05, |log_2_[FC] | ≤ −1 for colon or ≥ 1 for ileum). (E) Correlation plots between log_2_ fold changes in mRNA expression and corresponding cytokine secretion in ileum (blue) or colon (red).

Filgotinib was initially titrated *ex vivo* in non-CD-derived PCIS (1-100 nM) and showed no toxicity after 24 h of cultivation (Figure S7A and B). Dose-dependent inhibition of 19 mediators revealed variable IC_50_ values, with T cell-associated cytokines showing IC_50_ values within the tested range. Although several mediators responded at lower concentrations, 100 nM represented the lowest concentration that consistently affected a broader spectrum of T cell-associated mediators and was therefore selected for subsequent experiments (Figure S7C). In CD-derived PCIS from the ileum and colon, immune cell activation was induced with ConA, followed by treatment with 100 nM filgotinib for 24 h (Figure 3A). This treatment did not affect tissue viability (Figure 3B). Quantification of 20 inflammatory mediators revealed that filgotinib suppressed ConA-induced release of 15 mediators in both ileal and colonic PCIS supernatants (Figure S8A and B). Thus, filgotinib exerted anti-inflammatory activity in both intestinal regions. However, the magnitude and breadth of inhibition differed markedly between ileal and colonic PCIS. While several mediators were reduced in both regions, five key mediators associated with Th1/Th17 and cytotoxic immune responses showed preferential or substantially stronger inhibition in colonic PCIS. Specifically, no inhibition was observed for IL-17F (log_2_FC: −0.09 | −6.03; ileum | colon), IL-22 (log_2_FC: −0.15 | −10.08), and TRAIL (log_2_FC: −0.35 | −2.9), while IL-21 (log_2_FC: −2.05 | −5.6) and CXCL10 (log_2_FC: −0.7 | −6.7) were only partially inhibited in ileal compared with colon CD-derived PCIS (Figure 3C). To determine whether the mediator-level effects were reflected transcriptionally, we focused on the genes corresponding to the five mediators showing preferential or stronger suppression in colonic PCIS at the protein level. This targeted gene-level analysis showed that *IL17F*, *IL22*, *TNFSF10* (gene coding for TRAIL protein), and *CXCL10* were downregulated by filgotinib predominantly in colonic PCIS, whereas corresponding gene regulation in ileal PCIS was absent or did not reach statistical significance (Figure 3D; Table S6). To assess the concordance between mediator release and corresponding mRNA regulation, correlation analyses were performed separately for ileal and colonic PCIS. This analysis confirmed that the stronger mediator-level suppression in colonic PCIS was paralleled by corresponding transcriptional regulation, whereas ileal responses were weaker or less consistent (Figure 3E). Together, these mediator- and gene-level data identify the first major regional difference in filgotinib responsiveness: colonic PCIS showed stronger suppression of selected Th17-, Th1- and cytotoxicity-associated inflammatory mediators, whereas corresponding responses in ileal PCIS were weaker, partial, or absent.

**Figure 4.**
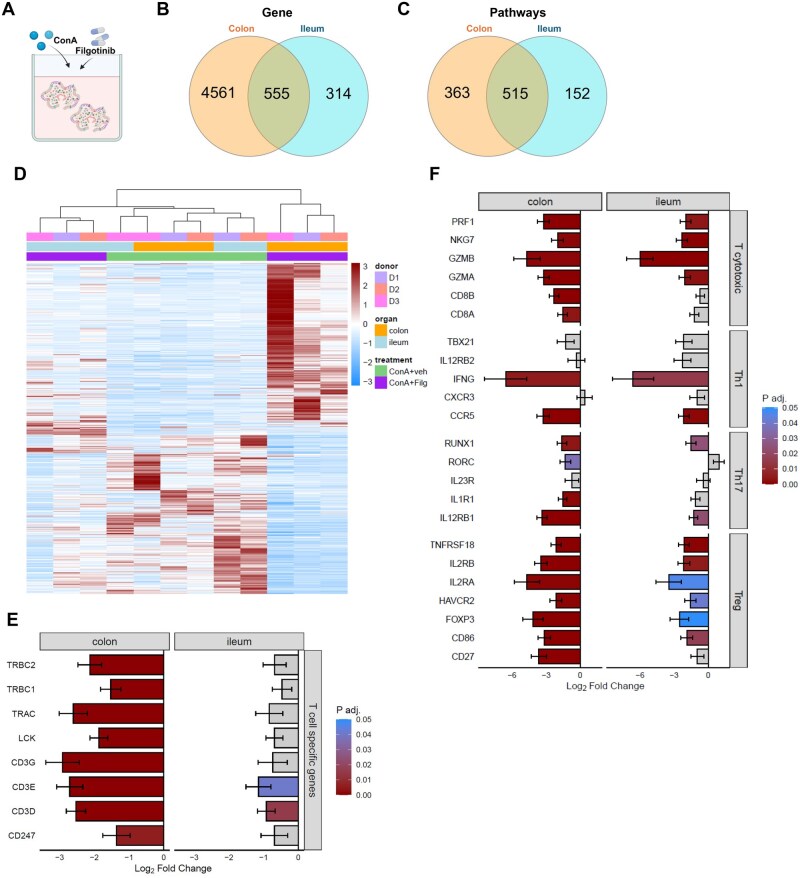
Filgotinib modulates T cell-associated transcriptional programs in a region-specific manner. Colon or ileum Crohn’s disease (CD)-derived precision-cut intestinal slices (PCIS) were incubated for 24 h either unstimulated (Med) or stimulated using 10 μg/mL Concanavalin A combined with 100 nM filgotinib (ConA + Filg) or 0.05% DMSO vehicle control (ConA + Veh). Upregulation of gene signature between PCIS from colon (*N* = 3) and ileum (*N* = 3) samples from CD patients was analyzed by mRNA sequencing. (A) Schematic representation of the treatment (created with BioRender.com). (B-C) Venn diagrams highlighting the overlap of all differential regulated genes (B; adj. *P* < .05) and all pathways (C), by comparing ConA + Veh and ConA + Filg in colon and ileum CD-derived PCIS. (D) Heatmap displaying expression levels of differentially expressed genes (DEGs; adj. *P* < .05, |log_2_[FC] | ≥ 1) among investigated groups. It shows the global transcriptome-level response to filgotinib and includes all DEGs identified after comparison of ConA + Filg vs ConA + Veh in ileal and/or colonic CD-derived PCIS according to the predefined statistical criteria. Genes are classified as shared, colon-specific or ileum-specific based on their significance in the respective regional comparison. The complete list of DEGs is provided in Table S6. Hierarchical clustering based on Euclidean distance. (E-F) Selected gene-level analysis of T cell receptor signaling and T cell subset-associated transcriptional programs for ConA + Veh vs ConA + Filg treated CD-derived PCIS from colon (*N* = 3) or ileum (*N* = 3). Genes were selected to illustrate immune programs identified within the broader filgotinib-regulated transcriptomic response. The corresponding pathway-level analysis is shown in Figure 5A.

**Figure 5.**
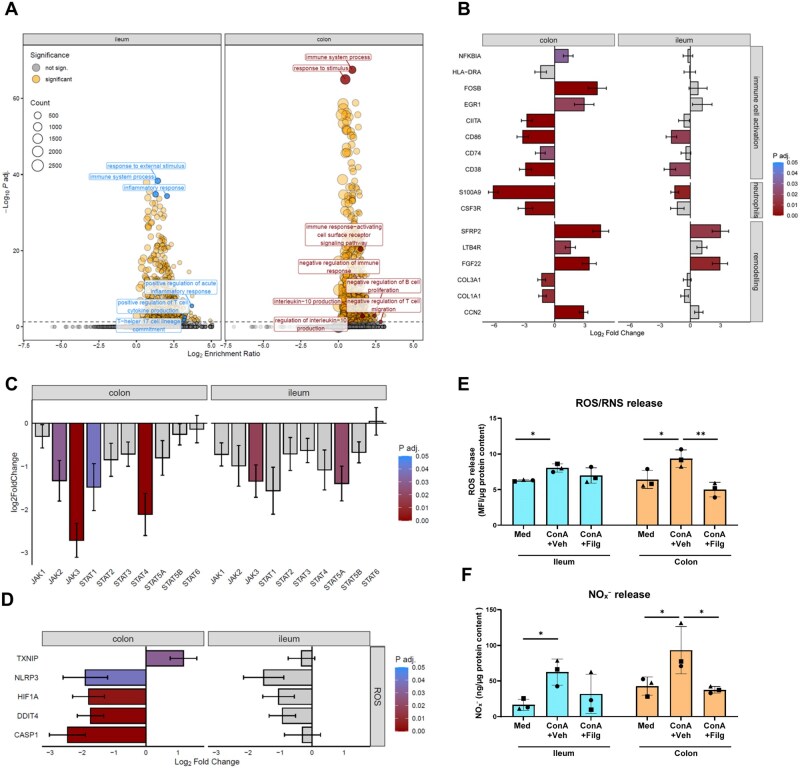
Filgotinib preferentially suppresses immune activation and oxidative stress responses in the colon vs ileum. Colon or ileum Crohn’s disease (CD)-derived precision-cut intestinal slices (PCIS) were incubated for 24 h either unstimulated (Med) or stimulated using 10 μg/mL Concanavalin A combined with 100 nM filgotinib (ConA + Filg) or 0.05% DMSO vehicle control (ConA + Veh). Upregulation of gene signature by mRNA sequencing and reactive oxygen/nitrogen species (ROS/RNS) as well as nitrogen oxides (Nox) (sum of nitrate [NO_3_^−^] and nitrite [NO_2_^−^] release measurements were performed. Analysis between PCIS from colon (*N* = 3) and ileum (*N* = 3) samples from CD patients were performed. (A) Over-representation analysis of filgotinib-regulated gene sets in ConA-stimulated ileal (blue) and colonic (red) CD-derived PCIS. This pathway-level analysis provides the functional context for the selected T cell-associated gene-level changes shown in Figure 4E and F. Complete pathway enrichment results, including pathway names, database sources, contributing genes, *P-*values and FDR-adjusted *P-*values, are provided in Table S7. (B–D) Gene expression levels of selected genes for ConA + Veh- vs ConA + Filg-treated CD-derived PCIS from colon (*N* = 3) or ileum (*N* = 3). (E) ROS/RNS release in PCIS supernatants after stimulation. MFI was normalized to total protein content. (F) Release of nitrate and nitrite in PCIS supernatant after stimulation was added up to the total NO (NOx) and normalized to total protein content. *N* = 3 donors each. *P < .05 and **P < .01 by two-way ANOVA with Sidak’s multiple comparison test comparing ConA + Veh vs Med, ConA + Veh vs ConA + Filg, or ileum vs colon.

Having established that selected inflammatory mediators were more strongly suppressed in colonic PCIS, we next asked whether this differential pharmacodynamic response was reflected by broader region-specific transcriptional regulation. We therefore compared the transcriptomic effects of filgotinib in ConA-stimulated ileal and colonic CD-derived PCIS. While Figure 3D focused on selected genes corresponding to the key inflammatory mediators quantified at the protein level, we next analyzed the global transcriptomic response to filgotinib. This broader analysis included all genes that met the predefined criteria for differential expression in the comparison of ConA + filgotinib versus ConA + vehicle in ileal and/or colonic PCIS. Transcriptomic analysis identified 5430 significantly regulated genes, of which 555 were shared, 4561 were colon-specific, and 314 were ileum-specific (Figure 4B and D; Table S6). Pathway enrichment analysis revealed 363 colon-specific, 152 ileum-specific, and 515 shared pathways affected by filgotinib treatment (Figure 4C; Table S7). To link the global filgotinib-induced transcriptomic response to defined immune mechanisms, we first examined selected genes representing T cell receptor signaling and T cell subset-associated programs (Figure 4E, F). These gene-level analyses were intended to illustrate key immune programs within the broader filgotinib-regulated transcriptome. The corresponding pathway-level analysis of filgotinib-responsive gene sets is presented in Figure 5A and was subsequently integrated to define the broader functional context of these gene-level changes. Filgotinib significantly downregulated several genes involved in T cell receptor (TCR) signaling, including TCR chains (*TRAC*, *TRBC1*, *TRBC2*), components of the CD3 signaling complex (*CD3D*, *CD3E*, *CD3G*, *CD247*), and the gene for proximal signaling kinase *LCK*, particularly in colonic PCIS than in ileal PCIS (Figure 4E). Further transcriptional analysis of T cell subsets revealed additional region-specific gene regulation. The Th17-associated genes *RUNX1*, *RORC*, *IL1R1*, and *IL12RB1* showed greater downregulation in colonic PCIS, while the downregulation of the Th1-associated genes *TBX21*, *IL12RB2*, *IFNG*, and *CCR5*, cytotoxic T cell-related genes *PRF1*, *GZMB*, *GZMA*, and *EOMES*, as well as regulatory T cell-associated genes (e.g., *FOXP3*, *IL2RA*, *IL2RB*, *HAVCR2*, and *TNFRSF18*), was comparable between ileal and colonic CD-derived PCIS *ex vivo* (Figure 4F).

These data identify the second major regional difference in filgotinib responsiveness: compared with ileal PCIS, colonic PCIS showed a broader transcriptional response to JAK1 inhibition, with more extensive regulation of genes and pathways linked to T cell activation and Th17-associated inflammatory programs.

Importantly, Figure 4D also revealed a substantial set of upregulated genes after filgotinib treatment, particularly in colonic PCIS. We therefore interpret the colonic response to filgotinib not only as suppression of T cell-associated inflammatory programs, but also as a broader transcriptional shift that includes induction of tissue-regulatory and repair-associated responses. This interpretation is supported by the subsequent pathway- and gene-level analyses, where filgotinib increased the expression of genes associated with tissue remodeling and repair, including *SFRP2*, *LTB4R*, *FGF22*, and *CCN2*, predominantly in colonic PCIS. Thus, the stronger transcriptional response to filgotinib in colonic tissue appears to involve both attenuation of inflammatory immune activation and induction of compensatory regulatory or repair-associated tissue programs.

### 3.4. Filgotinib reduces immune activation and oxidative stress more effectively in colonic vs ileal PCIS *ex vivo*

After identifying stronger mediator-level and transcriptional responses to filgotinib in colonic PCIS, we next assessed whether this regional difference extended to broader immune activation, JAK/STAT signaling and oxidative stress-associated tissue responses. Over-representation analysis of filgotinib-regulated gene sets was therefore performed to define broader region-specific functional programs associated with JAK1 inhibition in ileal and colonic CD-derived PCIS. It revealed distinct regional pathway responses to filgotinib treatment. In colonic PCIS, anti-inflammatory pathways including IL-10 production, negative regulation of B cell proliferation, and negative regulation of T cell migration were enriched. In contrast, ileal PCIS showed enrichment of pro-inflammatory pathways such as positive regulation of acute inflammatory response and positive regulation of T cell cytokine production (Figure 5A; Table S7). To illustrate these pathway-level findings at the gene level, we next examined representative genes involved in immune activation and antigen presentation, including *HLA-DRA*, *CIITA*, *CD86*, *CD74*, and *CD38*, as well as neutrophil-associated genes such as *S100A9* and *CSF3R*. These genes showed stronger downregulation in colonic PCIS, whereas regulation in ileal PCIS was weaker or absent. In contrast, the immediate early response genes *FOSB*, *EGR1*, and *NFKBIA* were selectively upregulated in colonic PCIS, suggesting persistence of regulatory and transcriptional adaptation programs despite suppression of proinflammatory pathways (Figure 5B). These selected genes were used to visualize representative components of the enriched functional programs, while pathway-level conclusions were based on the formal enrichment analysis shown in Figure 5A and Table S7.

Filgotinib further increased the expression of genes associated with tissue remodeling and repair, including *SFRP2*, *LTB4R*, *FGF22*, and *CCN2*, predominantly in colonic PCIS *ex vivo*. Conversely, genes linked to extracellular matrix organization and collagen deposition (*COL3A1* and *COL1A1*) were downregulated in the colon, indicating a shift away from extracellular matrix deposition programs, whereas only minor regulation was observed in ileal PCIS *ex vivo* (Figure 5B). This repair-associated gene induction supports the interpretation that filgotinib induces a broader tissue-regulatory response in the colon, beyond suppression of inflammatory T cell-associated programs.

Filgotinib also modulated JAK–STAT pathway genes, significantly downregulating JAK2, JAK3, STAT1, and STAT4 in colonic PCIS, whereas in the ileum only JAK3 and STAT5A were significantly reduced after ConA + filgotinib treatment compared with the ConA + vehicle control *ex vivo* (Figure 5C). Due to the role of JAK–STAT signaling in regulating inflammatory and oxidative stress responses, we assessed functional ROS production in PCIS *ex vivo*. ConA stimulation led to a significant increase in the release of ROS/RNS and total nitrogen oxides (NOx, sum of nitrate and nitrite) in both ileal and colonic CD-derived PCIS *ex vivo*, compared with unstimulated controls. However, co-treatment with filgotinib significantly reduced ROS (ileum: 8.04 vs 6.9 MFI/µg, colon: 9.3 vs 5.0 MFI/µg; ConA + vehicle vs ConA + filgotinib) and NOx^−^ release levels (ileum: 62.6 vs 31.8 ng/µg, colon: 93.3 vs 37.6 ng/µg) only in colonic PCIS compared with the ConA + vehicle control (Figure 5D and E). Consistent with these functional findings, transcriptional analysis revealed region-specific regulation of oxidative stress-associated genes. In colonic PCIS, the antioxidative regulator *TXNIP* was significantly upregulated, whereas inflammasome- and ROS-associated genes including *NLRP3*, *CASP1*, *HIF1A*, and *DDIT4* were downregulated following ConA + filgotinib treatment. In contrast, no significant changes were observed in ileal PCIS *ex vivo*. (Figure 5F).

Together, these data show that the broader response to filgotinib in colonic PCIS extends beyond cytokine suppression and includes attenuation of inflammatory activation and oxidative stress pathways.

## 4. Discussion

In this study, we show that ileal and colonic CD tissue differ not only in their inflammatory baseline state, but also in their responsiveness to JAK1 inhibition. To assess local inflammatory processes directly in patient tissue, we used PCIS as an *ex vivo* platform. The PCIS model preserves complex tissue architecture, resident cell populations, and the local intestinal microenvironment, providing a physiologically relevant platform for mechanistic and pharmacological investigations.^36^^,^^42–44^ Using this approach, we were able to link region-specific immune programs to differential filgotinib responsiveness in human intestinal tissue *ex vivo*. In line with our previous work, which revealed preservation of disease-specific immune signatures in PCIS derived from IBD patients,^36^ this study extends these findings by systematically analyzing region-specific immune activity and drug responsiveness in CD-derived intestinal tissue *ex vivo*.

Chronic mucosal inflammation and epithelial barrier disruption are well-established hallmarks of CD.^45^^,^^46^ In our study, both ileal and colonic CD-derived PCIS exhibited markedly increased basal secretion of IL-6 and IL-8, which are both essential drivers of mucosal inflammation in CD and areconsistently elevated in inflamed mucosa and plasma of patients.^47–49^ Their serum levels have also been associated with predicting clinical response to therapy.^50^ Additionally, CD-derived slices exhibited a loss of tight junction architecture, providing further evidence that key pathophysiological features of CD were preserved in tissue slices *ex vivo*. Alterations in tight junction proteins, including occludin, have been reported in patients with IBD and are associated with impaired epithelial barrier integrity. Consistently, circulating occludin levels have been shown to differ between patients with CD or UC and healthy individuals, supporting the clinical relevance of tight junction remodeling in IBD.^51^^,^^52^ These characteristics provided the pathological context for the subsequent analysis of region-specific immune responses and filgotinib effects.

Ileal and colonic PCIS retained region-specific structural and transcriptional features *ex vivo*, including villus-associated absorptive programs in the ileum and crypt-associated epithelial, barrier, and microenvironmental programs in the colon. These preserved regional characteristics provide the anatomical and functional context for interpreting segment-specific immune responses and differential filgotinib responsiveness.

While transcriptional differences between ileum and colon are well established, their relevance here is that they provide a mechanistic basis for region-dependent drug responsiveness. Our data suggest that colonic CD-derived PCIS are characterized by stronger inducible T cell-associated and JAK/STAT-linked inflammatory programs, whereas ileal PCIS retain broader innate and mucosal inflammatory features. Because filgotinib targets JAK1-dependent cytokine signaling, these differences may explain why colonic tissue showed stronger suppression of Th1/Th17 and cytotoxic mediators, T cell-associated transcriptional programs, JAK/STAT-related genes, and oxidative-stress-associated responses compared with ileal tissue.

Although regional differences in CD affecting the ileum or colon have been described at proteomic and transcriptomic levels, the functional responsiveness of tissue-resident immune cells in these areas remains poorly understood.^10–12^ Our study addresses this gap by assessing immune activity in *ex vivo* ileal and colonic tissue slices from CD patients, revealing regionally distinct immune signatures. compared with the colon, PCIS from the ileum exhibited increased release of MIP-3α and GzmA, accompanied by elevated expression of genes including *CCL25*, *NLRP6*, and *IL1R1* already at baseline. *NLRP6* and *IL1R1* are associated with inflammasome activation, while CCL25 and MIP-3α are involved in immune cell recruitment, indicating an enhanced innate immune response in ileal CD-derived PCIS.^53^^,^^54^ In contrast, baseline gene expression patterns in colonic CD-derived PCIS were dominated by adaptive immunity. This included increased gene expression of *TNFSF10* and *GPR15LG*, as well as basal release of T cell-associated mediators such as IFN-γ, GM-CSF, and TRAIL. Notably, GPR15LG mediates T cell homing, and both IFN-γ and GM-CSF are characteristic Th1 cytokines.^55^^,^^56^  *TNFSF10*, which encodes for TRAIL that is commonly expressed by cytotoxic T cells was found to have a dual role in intestinal immunity.^57^^,^^58^ It can exhibit anti-inflammatory properties in conjunction with Tregs by inducing apoptosis and inhibiting T cell activation but, conversely, it can promote inflammation by activating NF-κB and proinflammatory cytokines, thereby exacerbating inflammatory processes.^59–62^ Taken together, our data suggest that, even at the basal level, CD-derived PCIS derived from the colon exhibit enhanced regulation of T cell activity compared with those derived from the ileum. This regional immune heterogeneity was further pronounced upon *ex vivo* immune stimulation. In particular, ConA stimulation of ileal PCIS resulted in a significantly greater release of proinflammatory cytokines, such as TNF-α and GM-CSF, compared with colonic PCIS. This was linked to the upregulation of a broader array of inflammatory pathways at the RNA level, including those associated with macrophage or other innate cell activation. Conversely, colonic PCIS, particularly following T cell receptor-specific stimulation, exhibited higher secretion of T cell-associated cytokines, such as IFN-γ and IL-17F, and enrichment of T cell-related pathways, including Th1 response and differentiation, compared with ileal PCIS. Notably, Th17-associated pathways were similarly upregulated in both ileal and colonic PCIS following *ex vivo* stimulation. Kong et al. performed single-cell analyses of biopsy samples to demonstrate the region-specific landscape of immune dysregulation in CD, which is characterized by an increased frequency of myeloid and stromal cell subtypes in the terminal ileum, contrasted with a higher frequency of epithelial cells and CD4+ T cells in the colon.^15^ Our data extend these analyses, providing direct evidence that CD-derived PCIS from colonic tissue exhibit higher T cell activity of different subtypes, while ileal PCIS show a strong innate immune response in addition to Th17 activity *ex vivo*. Importantly, this functional immune divergence provides a plausible mechanistic basis for the differential response to JAK1 inhibition observed between both intestinal regions.

Post-hoc analyses of clinical studies indicate that distinct cellular environments along the gastrointestinal tract contribute to differential drug responses in ileal vs colonic CD.^10^^,^^16^ Consistent with this concept, Kaboub et al. reported in human intestinal mucosal explants that JAK inhibition with tofacitinib suppressed inflammatory signaling more robustly in colonic UC tissue than in ileal CD tissue, indicating region-specific differences in responsiveness to JAK-targeted therapies.^63^ In general, medications appear more effective in isolated colonic inflammation than in ileal-affected CD.^17–19^ For example, metronidazole was more effective in treating colonic CD,^18^ and both metronidazole and ciprofloxacin, in combination with budesonide, were ineffective in treating isolated ileal disease.^19^ Similarly, an anti-TNF-α agent (certolizumab) appears to be more effective in treating colonic than ileal manifestations.^17^ This issue becomes particularly critical when a clinical trial fails to demonstrate the efficacy of a therapy, as occurred in the DIVERSITY study for filgotinib.^34^ Despite its success in treating UC, the limited efficacy of filgotinib observed in clinical CD trials may be consistent with region-dependent differences in JAK1-responsive inflammatory programs, which highlights the potential value of human tissue-based models for assessing segment-specific drug responses.^33^^,^^64^ Our data suggest that one reason may be that key inflammatory programs are not equally JAK1-dependent across intestinal regions. Accordingly, we observed region-specific differences in response to filgotinib in complex colonic and ileal tissue models *ex vivo*. Following ConA stimulation, filgotinib effectively inhibited the release of IL-21, IL-22, IL-17F, TRAIL, and CXCL10 in colonic PCIS. In contrast, this suppressive effect was either absent or markedly reduced in ileal CD-derived PCIS. IL-21, IL-22, and IL-17F are recognized markers of Th1/Th17 responses, with elevated levels found in the inflamed mucosa of patients with CD, indicating their critical role in driving and exacerbating intestinal inflammation.^65–68^ IL-17F mRNA levels have been found to be elevated in inflamed biopsies of CD, but not UC, patients.^65^ Furthermore, TRAIL, a marker of cytotoxic T cell activity, has been associated with complications in CD, including the development of fistulas and strictures.^69^^,^^70^ Modulation of oxidative-stress-associated pathways has previously been reported for JAK inhibitors, including in intestinal mucosal explant models, and may contribute to their anti-inflammatory activity. In line with these observations, filgotinib also affected oxidative stress responses in our CD-derived PCIS model. ConA stimulation increased ROS/RNS and NOx production in both ileal and colonic tissue, indicating that both regions were capable of mounting oxidative and nitrosative stress responses under inflammatory challenge. However, filgotinib significantly reduced ROS/RNS and NOx release predominantly in colonic PCIS, whereas this effect was absent or less pronounced in ileal tissue. This region-specific reduction was accompanied by upregulation of the antioxidative regulator *TXNIP* and downregulation of ROS- and inflammasome-associated genes, including *NLRP3*, *CASP1*, *HIF1A*, and *DDIT4*. Since oxidative and nitrosative stress contribute to mucosal injury, barrier dysfunction, and immune activation in CD,^71^^,^^72^ these findings suggest that the broader colonic response to filgotinib may include attenuation of oxidative tissue stress in addition to suppression of inflammatory cytokine signaling.

Our data provide direct functional evidence that key mucosal inflammatory responses in the ileum, including components of the Th1/Th17-associated program, may be at least partially JAK1-independent, resulting in reduced sensitivity to filgotinib in this intestinal region. Despite its limited impact on Th1/Th17-associated cytokines, filgotinib was not entirely ineffective in ileal CD-derived PCIS in this study. Upon ConA stimulation, it effectively inhibited the release of several proinflammatory markers, including TNF-α, IFN-γ, and IL-17A, in both ileal and colonic tissue slices. This aligns with transcriptional data demonstrating that CD-derived PCIS from the ileum led to downregulation of typical JAK1–STAT-dependent genes, such as *STAT1*, *IL-6*, *TNF* and *IFNG*,^32^^,^^73^ after *ex vivo* filgotinib treatment. Interestingly, filgotinib also led to a modest downregulation of several Treg-associated genes, including *FOXP3*, *IL2RA*, *IL2RB*, *CD27*, and *CD86*, in both ileal and colonic CD-derived PCIS *ex vivo*. These genes are critical for the development and efficient function of Tregs, which contribute to immune tolerance and homeostasis in the gut.^74^^,^^75^ In CD, functional Tregs are essential for restoring immune balance, limiting chronic inflammation and promoting healing of damaged intestinal mucosa, thereby preventing disease progression and complications.^76^^,^^77^ However, in contrast to the regulation of Treg-associated genes observed in both intestinal regions *ex vivo*, filgotinib induced a broader suppression of inflammatory activation pathways predominantly in colonic PCIS. This was reflected by the downregulation of genes involved in T cell receptor signaling, antigen presentation, and immune activation, together with reduced expression of neutrophil-associated genes. In addition to suppressing inflammatory T cell-associated programs, filgotinib induced a distinct set of upregulated genes in colonic PCIS. Together with the enrichment of anti-inflammatory pathways and the induction of genes associated with tissue remodeling and repair, this suggests that the stronger response of colonic tissue reflects a broader shift from active inflammatory signaling toward regulatory and potentially repair-associated tissue programs. This aspect may be particularly relevant for understanding why JAK1 inhibition appears more effective in colonic than ileal inflammatory settings. These findings indicate that while filgotinib modulates regulatory gene programs in both ileal and colonic tissue, its anti-inflammatory effects are more pronounced in the colon, where it additionally suppresses key pathways associated with both adaptive and innate immune activation.

In conclusion, our study reveals distinct, region-specific mucosal immune responses in CD with a more pronounced Th1/Th17 profile in the colon and a dominant Th17/innate signature in the ileum. These differences were accompanied by markedly broader anti-inflammatory effects of filgotinib in colonic than in ileal tissue, including suppression of pathways associated with T cell activation, antigen presentation, and neutrophil-associated responses, while several inflammatory mediators and oxidative stress responses remained less affected in ileal tissue *ex vivo*. These findings suggest that inflammatory pathways in ileal CD may be less responsive to JAK1 inhibition compared with those in the colon. Our study also supports the use of PCIS as a human *ex vivo* platform to investigate region-specific drug responses in intestinal inflammation.

## Supplementary Material

jjag101_Supplementary_Data

## Data Availability

The authors declare that all data supporting the findings of this study are available within the article and its Supplementary Information files or from the corresponding author on reasonable request.
