# Supplementary material for: Region-specific immune regulation determines differential response to JAK1 inhibition in Crohn’s disease ex vivo
Source: J Crohns Colitis. 2026 Jul 30;20(7):jjag101. doi: 10.1093/ecco-jcc/jjag101 (PMC13423239; doi:10.1093/ecco-jcc/jjag101)
Supplement: jjag101_Supplementary_Data [file jjag101_supplementary_data.zip › ECCO-JCC-2025-1425_R2_Supplemental_material.pdf]

Supplement material

Figure S1

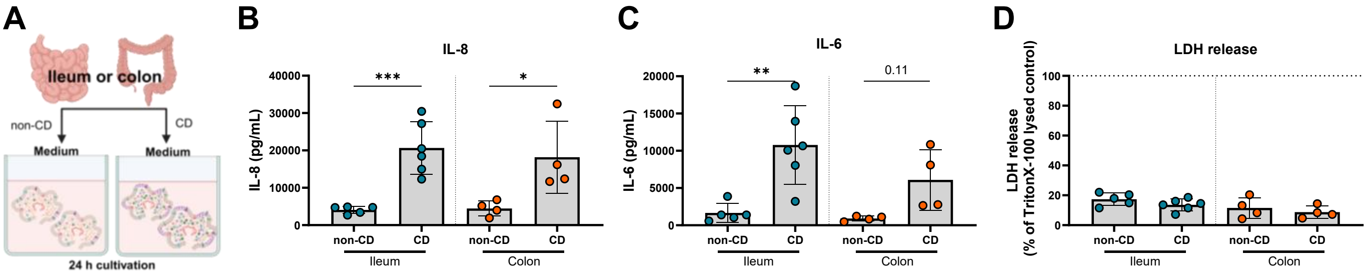

Figure S1 Basal inflammatory status and viability of ileal and colonic PCIS from CD and non-CD patients. Ileal and colonic PCIS from Crohn's disease (CD) and non-CD control patients were cultivated *ex vivo* in medium for 24 h. (**A**) Schematic representation of the experimental setup. (**B-C**) Basal secretion of the proinflammatory mediators IL-8 and IL-6 was quantified in culture supernatants. (**D**) LDH release was measured as an indicator of tissue viability and normalized to Triton X-100-lysed control tissue slices. N=4 donors each for CD and non-CD colon, N=5 non-CD ileum donors, and N=6 CD ileum donors. *p<0.05, **p<0.01, ***p<0.001 by two-way ANOVA with Sidak's multiple comparison test.

Figure S2

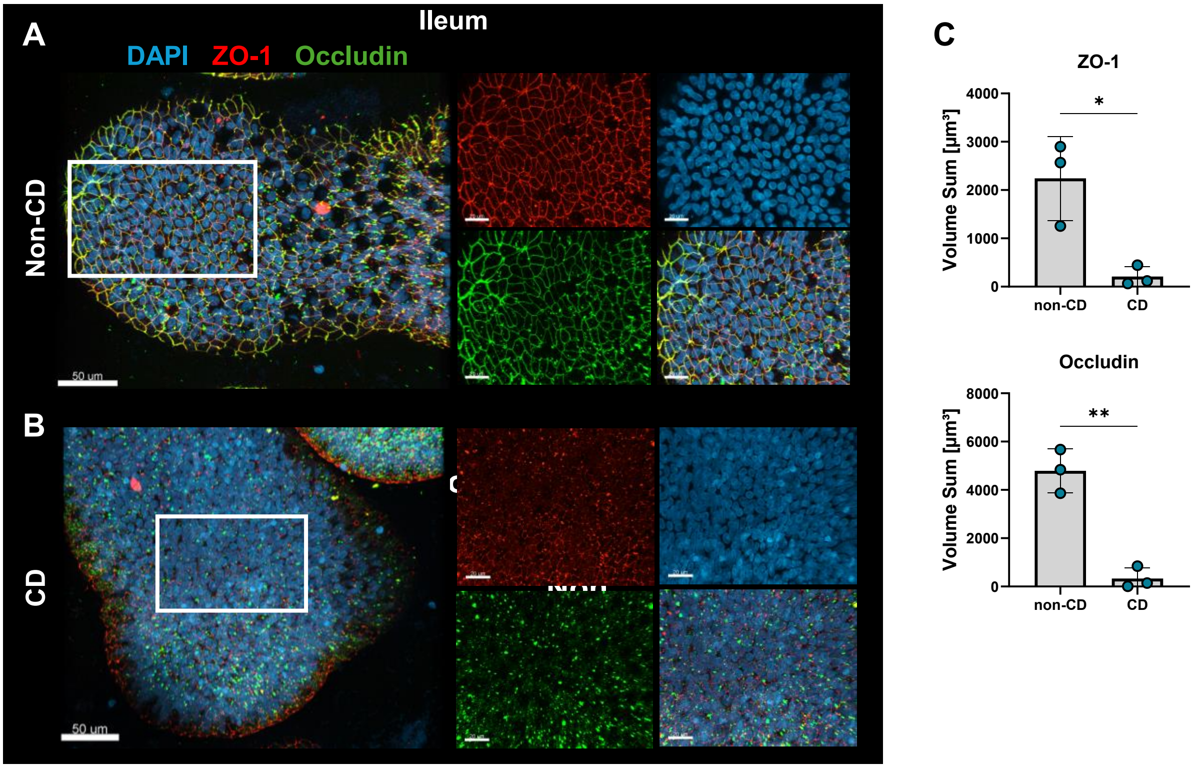

Figure S2 Epithelial integrity in ileum PCIS from CD and non-CD patients. Ileal PCIS from CD and non-CD patients were cultivated *ex vivo* in medium for 24 h. Whole-mount immunofluorescence staining was performed for the tight junction proteins occludin and ZO-1, together with DAPI nuclear staining. Representative images are shown for non-CD (**A**) and CD-derived ileal PCIS (**B**). (**C**) Quantification of ZO-1 and occludin staining intensity/volume in N=3 CD and N=3 non-CD donors. Reduced ZO-1 and occludin staining in CD-derived ileal PCIS indicates altered epithelial barrier architecture. *p<0.05, **p<0.01 by unpaired two-tailed t-test. Scale bars: 50 µm overview image and 20 µm magnified section.

Figure S3

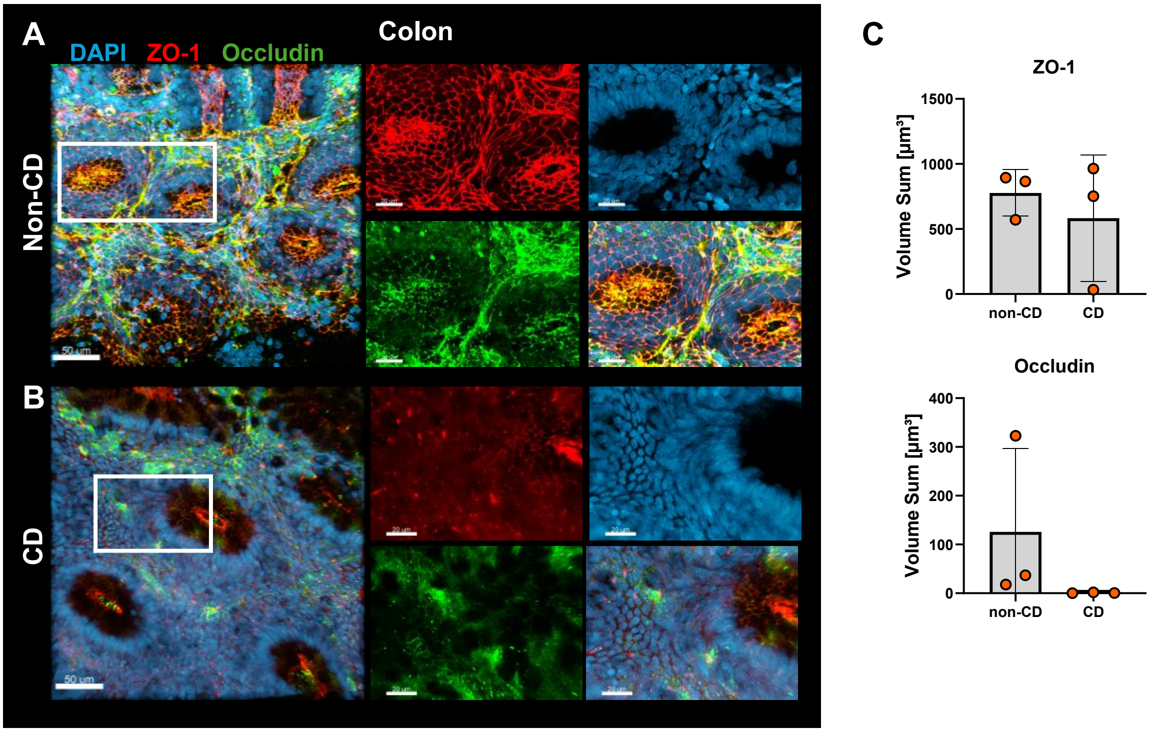

Figure S3 Epithelial integrity in colon PCIS from CD and non-CD patients. Colonic PCIS from CD and non-CD patients were cultivated *ex vivo* for 24 h. Whole-mount immunofluorescence staining was performed for occludin, ZO-1, and DAPI. Representative images are shown for non-CD (**A**) and CD-derived colonic PCIS (**B**). (**C**) Quantification of ZO-1 and occludin staining in N=3 CD and N=3 non-CD donors. No significant differences in ZO-1 or occludin staining were detected between CD- and non-CD-derived colonic PCIS under these conditions. Statistical analysis was performed using an unpaired two-tailed t-test. Scale bars: 50 µm overview image and 20 µm magnified section.

Figure S4

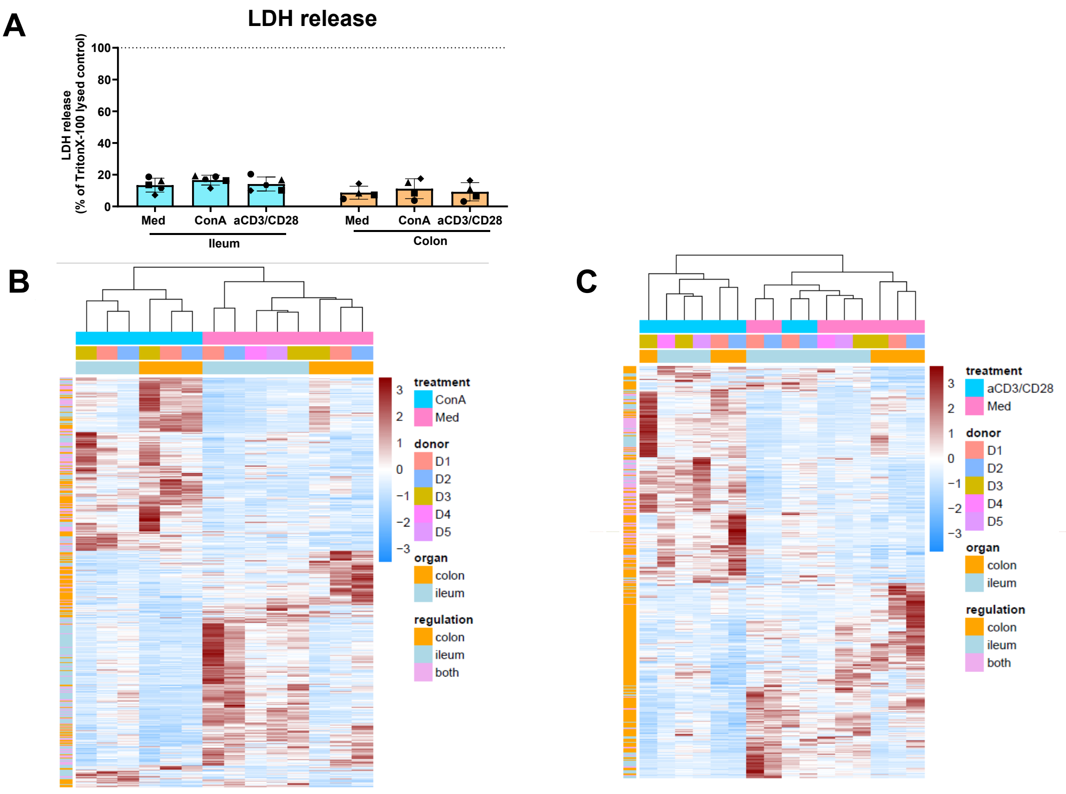

Figure S4 Effect of immunostimulatory treatment in CD-derived PCIS ex vivo. Colon or ileum tissue slices from CD patients were cultivated ex vivo either unstimulated (Med) or stimulated with 10 µg/mL Concanavalin A (ConA) or 10 µL/mL anti-CD3/CD28 antibodies. **(A)** LDH release in supernatant normalized to LDH release of Triton X-100 lysed control tissue slices. **(B+C)** Heatmap displaying expressions levels of differentially expressed genes (DEGs; adj. $p < 0.05$, $|\log2(FC)| \geq 1$) after ConA **(B)** or anti-CD3/CD28 stimulation **(C)**. Hierarchical clustering based on Euclidean distance.

Figure S5

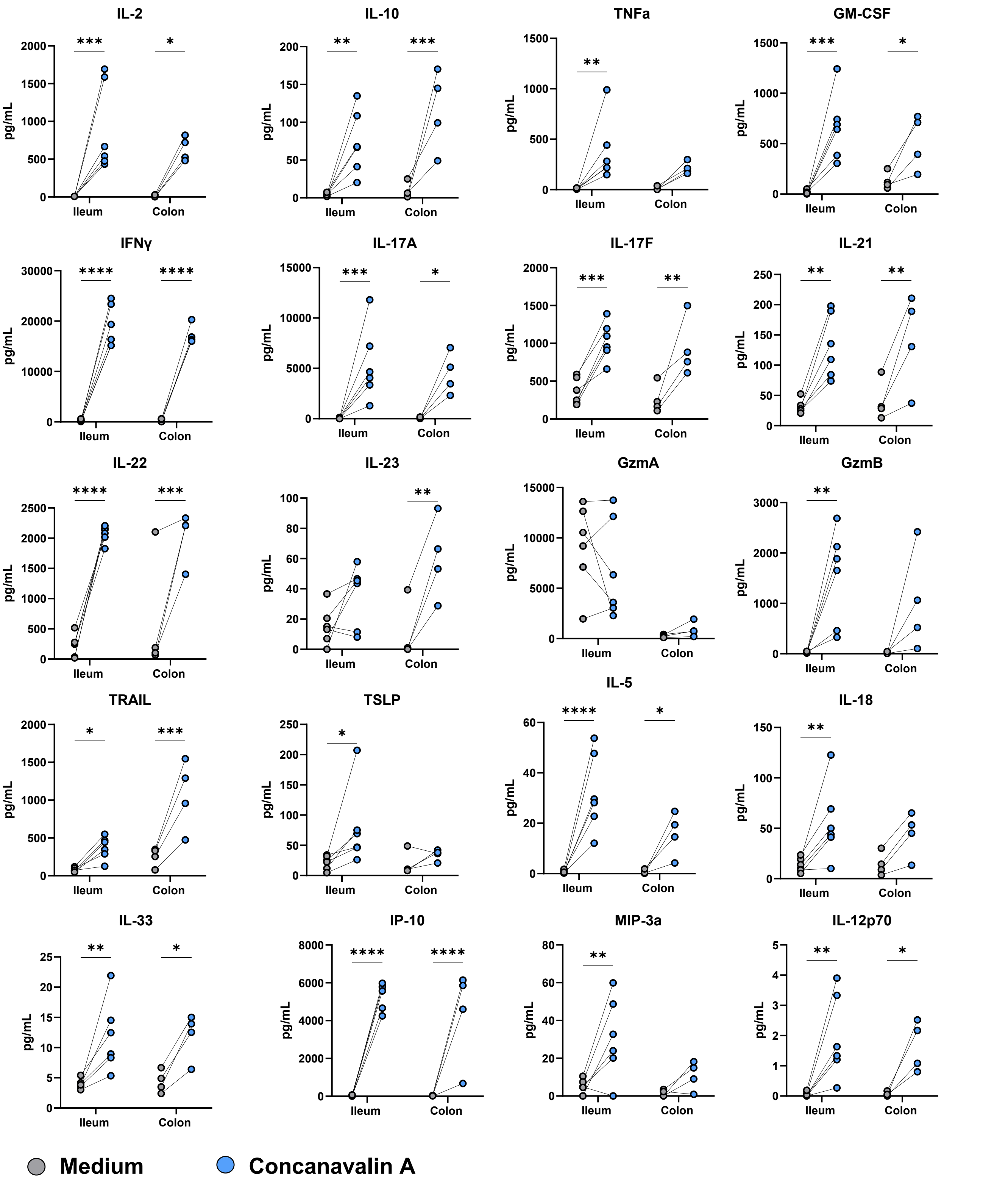

Figure S5 Amoun of metabolites released in CD-derived PCIS from colon and ileum following ConcanavalinA treatment. Colon or ileum CD-derived PCIS were cultivated ex vivo for 24 h either unstimulated (Med) or stimulated with 10 µg/mL Concanavalin A (ConA). Absolute release levels of 20 mediators in supernatant. *p<0.05, **p<0.01, ****p<0.0001 by two-way ANOVA with Sidak's multiple comparison test coparing Med. vs. ConA. N=4 colon and N=6 ileum donors.

Figure S6

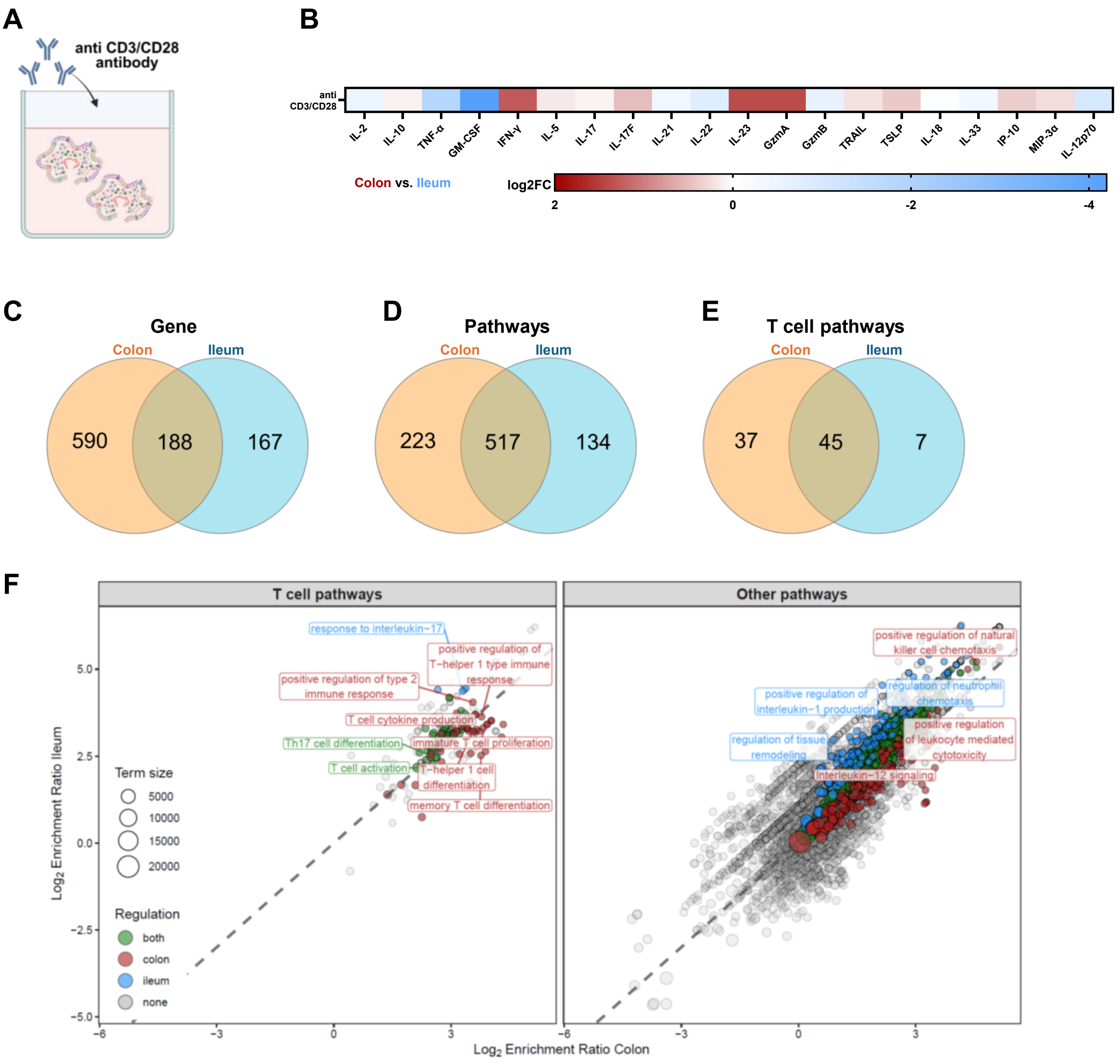

Figure S6 Differential activation of T cell signaling in terminal ileum versus ascending colon of CD patients ex vivo. Colon or ileum tissue slices from CD patients were cultivated ex vivo either unstimulated (Med) or stimulated with 10 µL/mL anti-CD3/CD28 antibodies (A). The secretion of several mediators in the supernatant as well as gene expression analysis were performed. (B) Released mediators levels as Log2 fold changes (ileum vs. colon) of stimulation-induced fold changes (medium (Med) vs. CD3/CD28 stimulation) in PCIS from CD patients. N=4 colon and N=6 ileum donors (log2FC, >0 values (red): higher in colon, <0 values (blue): higher in ileum). Per donor a minimum of two technical replicates (two wells with two tissue slices each) were analyzed. (C-F) Gene expression analysis after anti-CD3/CD28 antibody stimulation, comparing colon and ileum PCIS from CD patients. N=3 colon and N=5 ileum donors. (C-E) Veen-diagrams highlighting the overlap of all differential regulated genes (C), all pathways (D), and T cell pathways (E) by comparing colon and ileum CD-derived PCIS after CD3/CD28 stimulation. (F) Pathways enrichment plots of differentially regulated genes in colon vs. ileum PCIS for T cell pathways and all other pathways. Green: enriched in both, blue: colon-enriched, red: ileum-enriched.

Figure S7

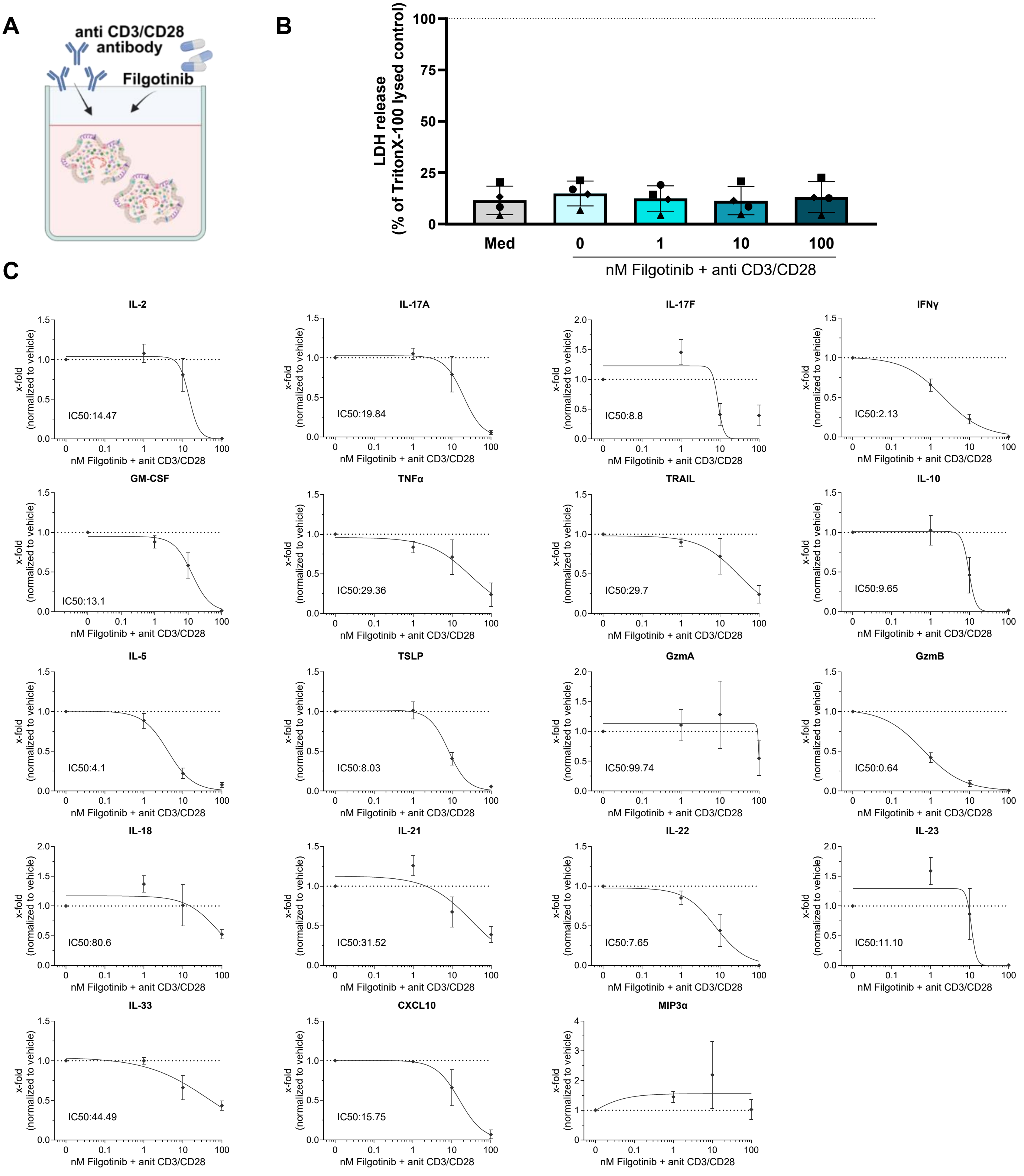

Figure S7 Dose-response curves of filgotinib treated PCIS from colon tissue ex vivo. PCIS derived from colon tissue of non-CD patients were utilized for a dose-response analysis of filgotinib ex vivo. T cell-specific stimulation was induced by treating PCIS with 10 µL of anti-CD3/CD28 without or with filgotinib at three concentrations (1, 10, 100 nM). **(A)** Schematic representation (created with BioRender.com). **(B)** LDH release in supernatant normalized to LDH release of Triton X-100 lysed control tissue slices. **(C)** Dose-response curves with IC50 values for 19 measured mediators. For each donor, released levels were normalized to anti-CD3/CD28 stimulation and shown as x-fold values. Mean±SD for N=4 donors.

Figure S8

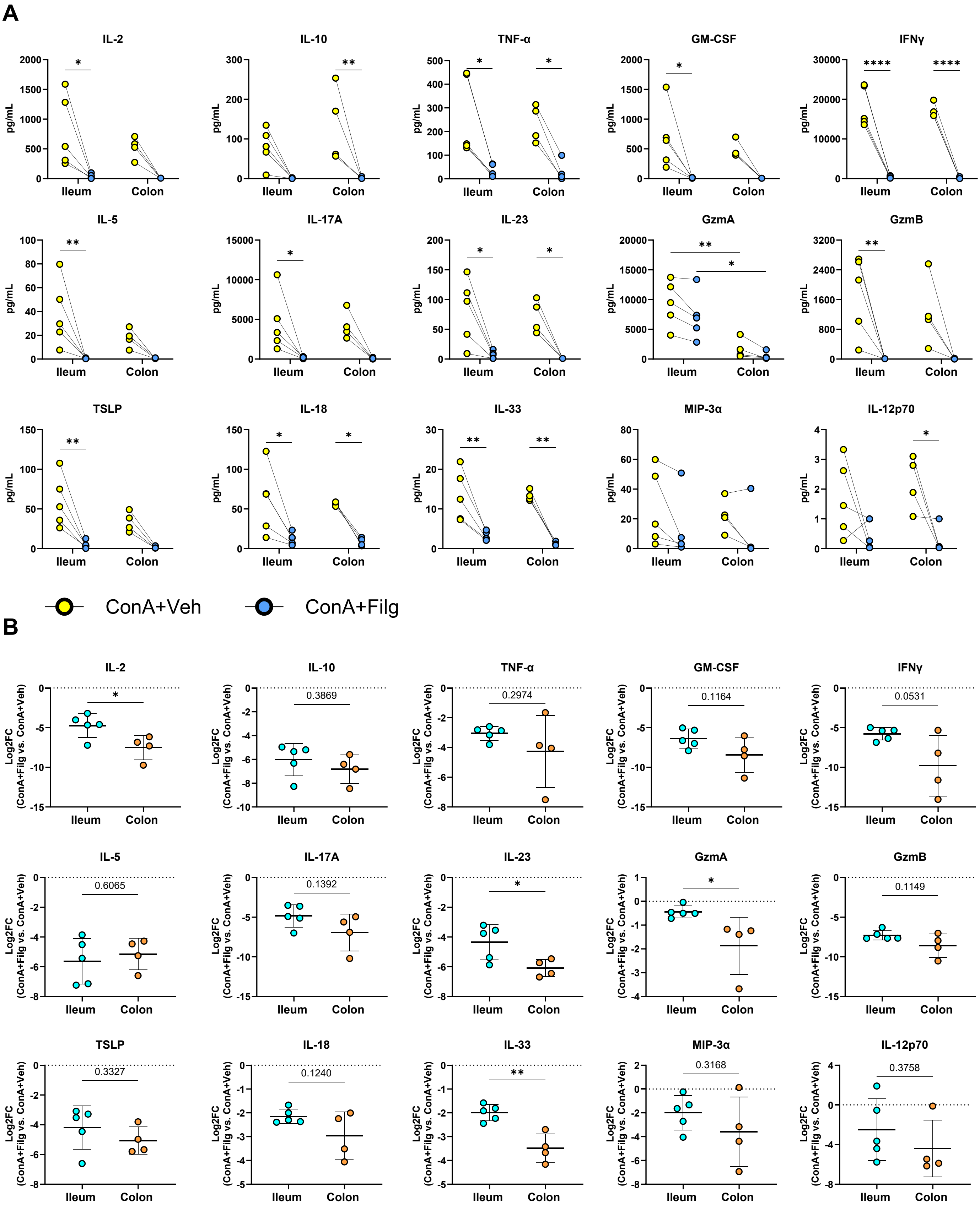

Figure S8 Additional metabolite release in CD-derived PCIS from colon and ileum following filgotinib treatment. Colon or ileum CD-derived PCIS were stimulated for 24 h using 10 µg/mL Concanavalin A combined with 100 nM Filgotinib (ConA+Filg) or 0.05% DMSO vehicle control (ConA+Veh). **(A)** Absolute release levels of further 15 mediators in supernatant. *$p<0.05$, **$p<0.01$, ****$p<0.0001$ by two-way ANOVA with Sidak's multiple comparison test comparing ConA+Veh vs. ConA+Filg or ileum vs. colon. **(B)** Log2FC representation of the ConA+Filg stimulated PCIS normalized to ConA+Veh. *$p<0.05$, **$p<0.01$, by unpaired two-tailed t-test comparing either colon vs. ileum. N=4 colon and N=5 ileum donors.

Figure S9

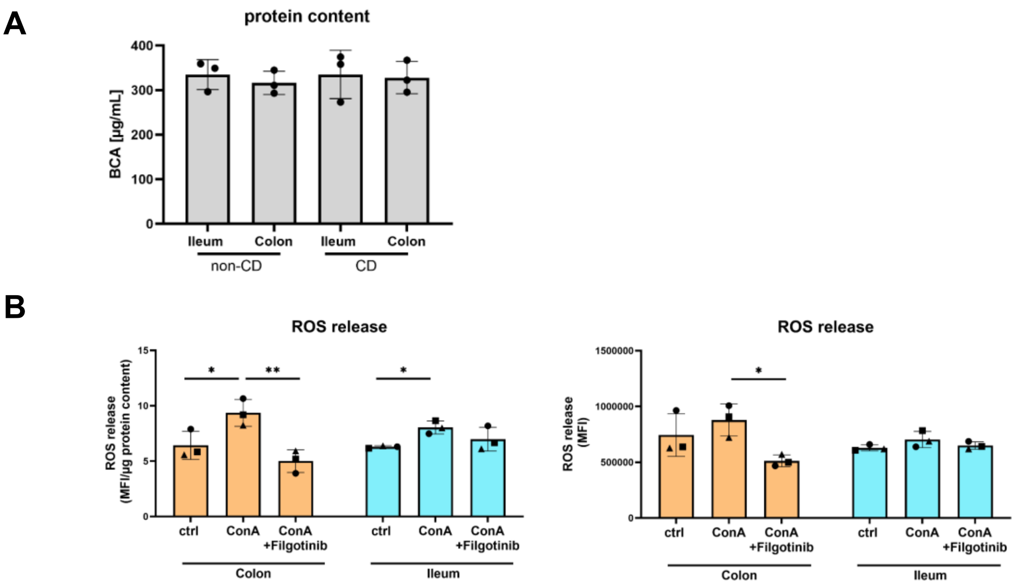

Figure S9 ROS/RNS raw data from colon and ileum following filgotinib treatment. Colon or ileum CD and non-CD-derived PCIS were stimulated for 24 h using 10 µg/mL Concanavalin A combined with 100 nM Filgotinib (ConA+Filg) or 0.05% DMSO vehicle control (ConA+Veh). **(A)** Protein content measured by BCA assay **B)** Fluorescence (MFI) data *p<0.05, **p<0.01, ***p<0.0001 by two-way ANOVA with Sidak's multiple comparison test comparing ConA+Veh vs. ConA+Filg or ileum vs. colon. N=3 colon and N=3 ileum donors.

Supplemental Tables:

Table S1 Extended patient information including diagnoses and medication history.

Table S2 Complete list of all enriched biological pathways based on overrepresentation analysis of colon and ileum PCIS from CD patients cultivated only in medium control for 24 h ex vivo. All samples were analyzed by mRNA sequencing with N=3 colon and N=5 ileum donors.

Table S3 Complete list of differential expressed genes (DEG) for colon and ileum PCIS from CD patients after 24 h ex vivo stimulation with 10 µg/mL Concanavalin A. All samples were analyzed by mRNA sequencing with N=3 colon and N=5 ileum donors.

Table S4 Complete list of enriched biological pathways based on overrepresentation analysis of DEGs in colon and ileum PCIS from CD patients after 24 h ex vivo stimulation with 10 µg/mL Concanavalin A. All samples were analyzed by mRNA sequencing with N=3 colon and N=5 ileum donors.

Table S5 Complete list of enriched inflammatory- and JAK/STAT-related biological pathways identified by overrepresentation analysis of DEGs in colon and ileum PCIS from CD patients after 24 h ex vivo stimulation with 10 µg/mL Concanavalin A. All samples were analyzed by mRNA sequencing with N=3 colon and N=5 ileum donors.

Table S6 Complete list of differentially expressed genes (DEGs) identified after comparison of PCIS stimulated for 24 h ex vivo with 10 µg/mL Concanavalin A combined with 100 nM Filgotinib versus 10 µg/mL Concanavalin A with 0.05% DMSO vehicle control in colon and ileum samples from CD patients. All samples were analyzed by mRNA sequencing with N=3 colon and N=3 ileum donors

Table S7 Complete list of enriched biological pathways based on overrepresentation analysis of DEGs in colon and ileum PCIS from CD patients after 24 h ex vivo stimulation with 10 µg/mL Concanavalin A and 100 nM Filgtoinib. All samples were analyzed by mRNA sequencing with N=3 colon and N=3 ileum donors.

Table S1 Extended informations about patient data, diagnoses, and medications

**Classification**	**Gender**	**Age [years]**	**Removed segment**	**Diagnosis**	**Histology / Findings**	**Home Medication**	**Prior Illnesses / Course**
non-CD	Male	78	terminal ileum	Coecum carcinom	not available	not available	Diabetes mellitus type II, arterial hypertension, hypercholesterolemia; only healthy tissue
non-CD	Male	76	terminal ileum	Colon carcinom	not available	not available	Arterial hypertension; only healthy tissue
non-CD	Female	75	terminal ileum	Colon carcinom	not available	not available	Parkinson's disease; only healthy tissue
non-CD	Female	64	transversum colon	Stenosis colon carcinom	Carcinoma in the cecum (pT3) & transverse colon (pT2)	Amlodipine, Ramipril, Amitriptyline, Dronabinol	Arterial hypertension; colonoscopy unremarkable
non-CD	Female	76	terminal ileum &ascending colon	liver cirrhosis, Coloncarcinom	not available	not available	Only healthy tissue at the resection margin; renal failure, arterial
non-CD	Female	58	terminal ileum &ascending colon	Ascending colon carcinom	not available	not available	Only healthy tissue at the resection margin; arterial hypertension
non-CD	Male	69	colon ascendence	Adenocarcinoma	not available	not available	Only healthy tissue
CD	Female	41	terminal ileum &ascending colon	Crohn’s disease	not available	Not known anti-inflammatory therapy	Not known pre-existing conditions except Crohn’s disease
CD	Male	62	terminal ileum &ascending colon	Crohn’s disease	not available	Not known anti-inflammatory therapy	Not known pre-existing conditions except Crohn’s disease
CD	Female	62	neuterminal ileum & ascending colon	Crohn’s disease/Terminal ileitis with ileus symptoms	Ulceration, transmural inflammation, granulomas, chronic-fibrosing	Dexamethasone paused, Cotrim, Ramipril, Bisoprolol, Dapagliflozin, Dekristol, Kalinor, Zoledronate, Aciclovir paused, Pentasa	Multiple myeloma IgG-Kappa
CD	Female	40	terminal ileum	Crohn’s disease/Terminal ileitis	Chronic fibrosing serositis, fistulas, granulation tissue, plasma cell-rich inflammation	Prednisolone, Pantoprazole, Modulen	none
CD	Male	46	terminal ileum &ascending colon	Abscess conglomerate in Crohn’s disease	Conglomerate, fistulas, abscessing inflammation,	Pip/Tazo, Meropenem, Upadacitinib, Pantoprazole	None; colonoscopy: inflammatory stenosis
CD	Female	29	terminal ileum	Therapy-refractory Crohn’s colitis	Discontinuous inflammation, fistulas, granulomas up to the resection margin	Modulen, Cortiment, Dekristol, Folic acid	C. diff 2010, thyroid disease, liver/kidney, recurrent UTIs, long therapy course
